# Rapid Birth-and-Death Evolution of Imprinted snoRNAs in the Prader-Willi Syndrome Locus: Implications for Neural Development in Euarchontoglires

**DOI:** 10.1371/journal.pone.0100329

**Published:** 2014-06-19

**Authors:** Yi-Jun Zhang, Jian-Hua Yang, Qiao-Su Shi, Ling-Ling Zheng, Jun Liu, Hui Zhou, Hui Zhang, Liang-Hu Qu

**Affiliations:** 1 Key Laboratory of Gene Engineering of the Ministry of Education, State Key Laboratory of Biocontrol, and School of Life Sciences, Sun Yat-sen University, Guangzhou, P. R. China; 2 Laboratory of Liver Disease Hospital, The Third Affiliated Hospital, Sun Yat-sen University, Guangzhou, P. R. China; 3 Institute of Human Virology, Zhongshan School of Medicine, Sun Yat-sen University, Guangzhou, P. R. China; CNRS, France

## Abstract

Imprinted small nucleolar RNAs (snoRNAs) are only found in eutherian genomes and closely related to brain functions. A complex human neurological disease, Prader-Willi syndrome (PWS), is primarily attributed to the deletion of imprinted snoRNAs in chromosome 15q11-q13. Here we investigated the snoRNA repertoires in the PWS locus of 12 mammalian genomes and their evolution processes. A total of 613 imprinted snoRNAs were identified in the PWS homologous loci and the gene number was highly variable across lineages, with a peak in Euarchontoglires. Lineage-specific gene gain and loss events account for most extant genes of the HBII-52 (SNORD115) and the HBII-85 (SNORD116) gene family, and remarkable high gene-birth rates were observed in the primates and the rodents. Meanwhile, rapid sequence substitution occurred only in imprinted snoRNA genes, rather than their flanking sequences or the protein-coding genes located in the same imprinted locus. Strong selective constraints on the functional elements of these imprinted snoRNAs further suggest that they are subjected to birth-and-death evolution. Our data suggest that the regulatory role of HBII-52 on 5-HT_2C_R pre-mRNA might originate in the Euarchontoglires through adaptive process. We propose that the rapid evolution of PWS-related imprinted snoRNAs has contributed to the neural development of Euarchontoglires.

## Introduction

**Figure 1:**
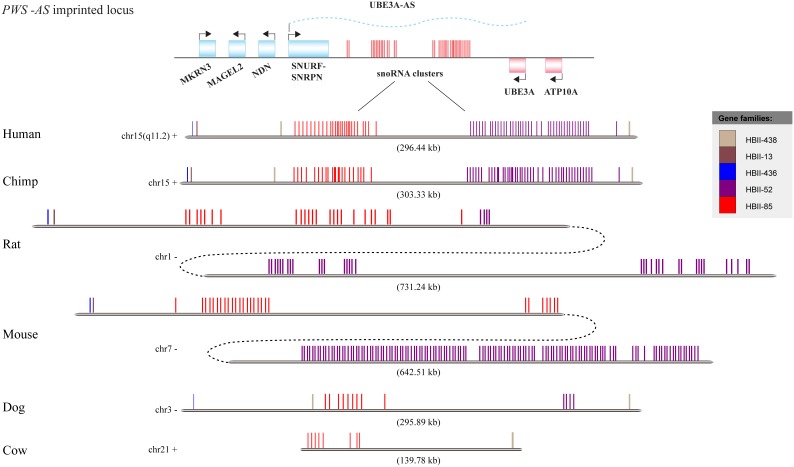
A schematic diagram of the imprinted snoRNA gene cluster in the PWS-related imprinted locus. The top panel depicts the gene distribution across the entire PWS locus. Pink represents maternally expressed genes, blue represents paternally expressed genes, red represents imprinted snoRNA clusters, and the dashed wavy line represents non-coding transcripts. In the lower panels, each snoRNA gene is represented by a vertical bar. The distribution of snoRNA genes was scaled to their genomic location, and the colors indicate their respective gene families. Clusters are scaled relative to their size within the chromosome.

Small nucleolar RNAs (snoRNAs) belong to a large group of small, metabolically stable RNAs that primarily direct the post-transcriptional modification of ribosomal RNA (rRNA) nucleotides. Based on common sequence motifs involved in the assembly of sno-ribonucleoprotein (snoRNP) particles, snoRNAs fall into two major classes, the C/D box and H/ACA box groups, which account for the 2′-O-ribose methylation and pseudouridylation modifications respectively of rRNA or snRNA [1], [2]. These snoRNAs are called ‘guide’ snoRNAs, and there is a fraction of snoRNAs, the so called ‘orphan’ snoRNAs, which do not target to any rRNA or snRNA. In recent years, hundreds of snoRNAs have been discovered in imprinted regions of the eutherian genomes (imprinted snoRNAs) [3]–[8]. The expressions of imprinted genes are depending on the parent of origin. Unlike other snoRNAs, which show broad expression profiles, imprinted snoRNAs are mainly expressed in the brain and are closely associated with brain function [9], [10]. Another feature of imprinted snoRNAs is that they usually fall within large gene clusters containing several gene families. One of such family usually contains dozens of snoRNA genes. Two large paternally expressed C/D box snoRNA families, HBII-85 (SNORD116) and HBII-52 (SNORD115), are located in the 15q11-q13 imprinted region of the human genome. Large interstitial deletions of this region underlie ∼70% of cases of Prader-Willi syndrome (PWS, [MIM176270]), a complex human neurological disease [11]. Duplication of the same region is the only recurrent cytogenetic aberration associated with autism, occurring in up to 5% of autism cases [12]–[14]. The clinical symptoms of PWS include neonatal hypotonia, feeding difficulties and failure to thrive during infancy, excessive weight gain after 18 months, hyperphagia, hypogonadism, global developmental delay and equivocal facial features. Deletion of the HBII-85 snoRNA cluster results in an exhibition of all major clinical symptoms of PWS in humans [15]–[17], but the role of the HBII-52 cluster in PWS has been difficult to define [11], [18]. The neuronal-specific HBII-52 snoRNAs have been reported to participate in the post-transcriptional regulation of the pre-mRNA encoding the 5-hydroxytryptamine 2C receptor (5-HT_2C_R), an important neurotransmission protein, including A-to-I RNA editing and alternative RNA splicing, with *in vitro* experiments [19]–[21]. The biological importance of the regulatory role of HBII-52 *in vivo*, if any, remains to be demonstrated. Knockout of MBII-52 (murine homologues of HBII-52) in a murine model led to an increase in the editing of 5-HT_2C_R pre-RNA and alterations in a number of 5HT_2C_R-related behaviors, including impulsive responses, locomotor activity and reactivity to palatable food [22]. Although the molecular targets of HBII-85 are as yet unknown, an increasing number of studies have investigated the regulatory roles of HBII-85 snoRNAs and their connection to PWS phenotypes [15]–[17], [23], [24]. In addition, the PWS locus contains several singleton snoRNA genes of unknown function, e.g., HBII-438, HBII-13 and HBII-436 (Figure 1).

SnoRNA genes have traditionally been regarded as conservative components of the genome. SnoRNAs have been identified in a wide spectrum of organisms spanning eukaryotes to archaebacteria, suggesting that they represent an evolutionary ancient group of non-coding RNAs [25]. Homologues of almost all yeast snoRNAs can be found in the human genome [26], [27], and their modification sites in rRNAs are also highly conserved among distantly related eukaryotes [1], [28]. However, the discovery of a large number of eutherian-specific imprinted snoRNAs led us to reexamine this supposedly ancient group of RNAs from a new perspective. Further investigations failed to find homologs of these imprinted snoRNAs in any other non-eutherian mammals or vertebrates [29]–[31]. In addition, a number of lineage-specific snoRNA genes were identified only in rodents rather than humans, and a rat-specific imprinted snoRNA cluster, RBII-36, containing 74 member genes, was not detected in mice (see the review of [32]). These observations suggest that imprinted snoRNA genes represent a class of newly derived small non-coding RNAs that might be subjected to rapid evolution.

Although an increasing number of imprinted snoRNAs have now been identified, they are mainly limited to a few model species, including human, mouse and rat. Since the PWS-related imprinted snoRNAs are closely associated with mammalian brain functions, it is of great interest to investigate the evolution of these highly dynamic non-coding RNAs. Nahkuri et al. suggest that the HBII-52 gene family originates from a non-imprinted snoRNA, SNORD119 [33]. Ogorelkova et al. revealed that the HBII-52 family was subjected to positive selection in Europeans populations, suggesting these gene are still undergoing evolution in human populations [34]. However, these studies did not fully reveal the evolutionary process of these imprinted snoRNA genes along with the differentiation of the eutherian or its significance to mammalian neural development. In this study, using a genome-wide snoRNA identification program developed by our group, snoSeeker [35], we performed comprehensive identification and evolutionary analyses to reveal the pattern and process underlying the evolution of mammalian imprinted snoRNAs.

## Materials and Methods

### Identification and Classification of Imprinted snoRNA Genes in Mammalians PWS Locus

The genome sequences and related whole genome alignments (WGAs) of 12 placental mammals (Human, Chimp, Rhesus, Rat, Mouse, Dog, Cat, Horse, Cow, Armadillo, Tenrec and Elephant) were downloaded from the UCSC genome website (http://genome.ucsc.edu/and http://genome-test.cse.ucsc.edu/). These species represent all major lineages of extant placental mammals. The range of human PWS locus was identified by the gene pair of MKRN3 and ATP10A. The homologous region of PWS in other species was obtained using the “covert” tool of the UCSC genome website and the gene content was used as an index when the result of covert tool is poor. The information of PWS loci in different species is listed in Table S1.

Two independent strategies were employed to search imprinted snoRNA candidates in the PWS loci: (1) The comparative genomics identification strategy with WGAs was performed as described in Yang et al. [35]. (2) The single-genome strategy was performed by searching the PWS homologous regions with snoSeeker program (http://202.116.74.192/snoSeeker/downloads.php) under the cutoff value of >24.0. The non-redundant candidates from these two strategies were combined for further gene family classification. The sequences of non-imprinted snoRNA genes of 15 vertebrates (with the addition of opossums, platypus and chicken) were identified from the WGAs of human and each of other species as described in Yang et al. [35]. All identified imprinted and non-impritned snoRNA gene sequences were provided in the Data S1.

For imprinted snoRNA gene family classification, BLAST search was first conducted to identify homologs to know human or rodents imprinted snoRNA genes, the cutoff is set to 10^−3^. Nevertheless, due to algorithmic characteristics and sensitivity constraint, this classification method will lead to ambiguities when dealing with the species that are distantly related to the reference. In addition, the genomes of some species were poorly assembled, e.g. the elephant and the tenrec genome, which could not provide accurate gene mapping information to validate the classification results. Thus, phylogenetic methods were further exploited to classify imprinted snoRNA candidates into known gene families. For this purpose, phylogenetic trees containing imprinted snoRNA candidates of each species were constructed with the human imprinted snoRNA genes as the reference for family classification. The threshold value of branch support for the assignment of a candidate to known gene families was >50%. The phylogenetic trees were constructed with MrBayes ver. 3.1 [36] with a HKY+G model.

### Phylogenetic Analyses

To reconstruct the phylogenetic relationship of imprinted snoRNA genes in the *PWS* locus, a dataset containing gene sequences from all families in this locus (i.e. HBII-436, HBII-13, HBII-438, HBII-85 and HBII-52) was generated. For gene families containing more than 20 members, 4–6 representative genes were selected based on a gene tree constructed using the neighbor-joining method implemented in MEGA ver. 3.1 [37]; for gene families containing less than 20 members, all sequences were added to the dataset. The sequences were aligned with ClustalX [38]. The sequence alignment is provided in data S1 and S2. Then a phylogenetic network was constructed using the NeighborNet method implemented in SplitsTree ver. 4.10 [39].

To further investigate the phylogenetic relationship within the HBII-52 and HBII-85 multi-gene families, the alignment of each family was built as described above. The phylogenetic relationships were reconstructed using MrBayes ver. 3.1 [36] with a HKY+G model suggested by MrModeltest (ver. 2.2) [40]. Every analysis consisted of two runs each containing four Markov chains that ran for 2×10^6^ generations, sampled every 1000 generations, with a random starting tree, default priors and equal branch lengths. The consensus trees were edited with iTOL [41]. The sequence alignments are provided in Data S2.

### Evolutionary Changes of the Number of Imprinted snoRNA Genes

To estimate the numbers of gene in ancestral species and gene gains and losses, we used the reconciled tree method [42]–[44], in which the topology of a gene tree is reconciled with that of a species tree. A simple example is shown by Niimura and Nei [45] and Nam and Nei [44]. To apply this method to imprinted snoRNA genes, we requested the computer program described in Niimura and Nei [45], [46] from Y. Niimura. We first constructed a phylogenetic tree using all genes of the two multi-gene families (HBII-52 and HBII-85) together with outgroup genes chosen from other close related families. The reconciled tree method was applied to these two phylogenetic trees and the Euarchontoglires tree topology. Given the relative low bootstrap value caused by short sequence of snoRNA genes, the 50% bootstrap condensed trees were used here.

To investigate the expansion process of imprinted snoRNAs, a combined physical-phylogenetic map for the HBII-52 or HBII-85 gene family of human or mouse was constructed. The phylogenetic trees were built with the Bayesian method as described above, each Markov chain ran for 1×10^7^ generations. The clades receiving >50% posterior probabilities were kept and the derived consensus trees were reconciled with gene physical locations in the chromosomes.

### Evolutionary Analyses

The number of new genes was calculated as follows. For imprinted snoRNAs, the number of newly derived genes was estimated according to the evolutionary change in gene number described above, whereas the number of newly derived non-imprinted snoRNAs was estimated by determining the difference in gene number between closely related species. Birth rates of new genes were calculated by dividing the number of newly derived genes by the lapsed time since the species diverged from their most recent common ancestor. The time scale was set according to Hedges et al. [47], and the weighted average data were selected.

For each imprinted gene family, the substitution rates between humans and each of the other species were calculated using the “between-group average calculation” method with the Kimura 2-parameter model implemented with MEGA ver. 3.1 [37], [48]. A “group” corresponds to a multi-gene family from one species, which contains all gene members in this family. For each between-group average, an arithmetic average is computed for all valid inter-group pairwise comparisons. The standard error for each calculation was estimated by bootstrap analysis with 500 replicates. To calculate the substitution rates of non-imprinted snoRNA genes, we first identified all of the families that were conserved between humans and each of the other species (Table S2, the sequences were provided in data S1 and S2). The guide snoRNAs and orphan snoRNAs were analyzed separately. The orphan snoRNA genes were identified by the absence of targeting sites in rRNA or snRNA according the information in snoRNA-LBME-db (https://www-snorna.biotoul.fr/index.php) [27]. These orphan snoRNAs were listed in Table S2. For multi-gene families, one gene member and its orthologous in the other species s was selected for the analysis. Then gene sequences from one species were linked together in order of gene name and an aliment was built with this integrated sequence of human and each of the other species. Next, the average substitution rates of the conserved families were calculated with the Kimura 2-parameter model. All of the calculations were performed using MEGA version 3.1 [37].

To test whether the rapid rate of evolution was specific to the imprinted snoRNAs, we compared the evolutionary rate between imprinted snoRNA genes and their flanking intergenic sequences. We obtained 500 bp of both upstream and downstream flanking sequences for each analyzed imprinted snoRNA gene from the UCSC genome website. The substitution rates of both imprinted snoRNA genes and their flanking sequences were calculated as described above. Similar analyses were also performed using non-imprinted snoRNA genes as the reference.

According to the secondary structure we divided box C/D snoRNs gene into 5 parts: the stem region, C/D boxes, antisense element 1 (ASE1), antisense element 2 (ASE2) and the remaining sequence. To investigate the evolutionary rates of different parts of the imprinted snoRNA genes, we created an alignment for HBII-52 or HBII-85 family and defined different structural components in the alignment. The evolutionary distances (*K*) between humans and each of the other species were calculated for each of the five parts of the snoRNAs using the Kimura 2-parameter model.

Tajima’s *D* test was performed with DnaSP software version 5.10.01[49]. For the Tajima’s neutrality test, significant deviations of Tajima’s *D* values from zero suggest rejecting the standard neutral model and negative values of Tajima’s *D* indicate positive selection. All the gene sequences of HBII-52 or HBII-85 gene family from one species were used for the test. The statistical significance was obtained assuming that *D* follows the beta distribution[50].

The K_a_/K_s_ or d_n_/d_s_ test is one of the most frequently used tests of selection. The test relies on the simple fact that the rate of evolution can exceed that of a neutral sequence only if there is positive selection [51]. Ka/Ks test for protein-coding gene was performed with MEGA 4.0 software [52].

### PWS Related snoRNA Targeting Pattern Analysis

To investigate the targeting pattern of HBII-52 gene families in different eutherians species, homologous sequence to human 5-HT_2C_R mRNA of different species were got by BALSTP program from ensembl genome web site (www.ensembl.org). The binding pattern between ASE2 sequences and 5-HT_2C_R mRNA in same species was analyzed by BLAST method with some modifications. Two major characters, the length of perfect binding and the distance of perfect binding from D box, were analyzed.

## Results

### Identification and Characterization of Imprinted snoRNAs in the PWS Homologous Locus of 12 Eutherian Genomes

Both single genomic and comparative genomic strategies were applied to identify imprinted snoRNA genes from 12 placental mammals to achieve high sensitivity and accuracy simultaneously (Table S1). To classify the snoRNA gene families, BLAST searches and phylogenetic analyses were combined to avoid the misassignment of individual gene members to known gene families. A total of 613 imprinted snoRNA genes were identified in the PWS homologous loci (the 15q11-q13 region of the human genome) of 12 eutherian genomes (Table 1, all snoRNA gene sequences were provided in Data S1). In general, primates and rodents have many more imprinted snoRNAs than other mammals, mainly reflected by the big volume of the HBII-52 and the HBII-85 multi-gene families. On average, primates have 77 PWS-related imprinted snoRNAs, rodents have 112, while Laurasiatheri and Xenarthra have only 17. The number of imprinted snoRNAs in elephants was comparable to that of primates. Certain gene families are missing from the ancestral lineages of placental mammals, such as Xenarthra and Afrotheria. For example, the HBII-85 gene family was missing from the tenrec genome, whereas HBII-52 was absent from the cow and the armadillo genomes. These observations may have two explanations: the first is due to the incomplete assembly of these genomes, the second one attributes to particular gene loss events occurred in these lineages. Conversely, extensive expansions of particular gene families have occurred in the mouse and elephant, leading to an excess of HBII-52 genes (130 and 70, respectively). The gene numbers of the multi-gene families (HBII-52 and HBII-85) predicted in this study were slightly lower than those of previous studies on the model species (e.g. human and mouse) [4], [5], [53]. It may attribute to the more stringent criteria for snoRNA identification used in the snoSeeker program [35].

**Table 1:** Statistics of imprinted snoRNA genes in the PWS locus of mammalians.

			HBII-436	HBII-13	HBII-438A	HBII-85	HBII-52	HBII-438B	Total
**Euarchontoglires**	**Primates**	**Human**	1	1	1	27	41	1	72
		**Chimp**	1	1	1	22	44	1	70
		**Rhesus**	1	1	1	28	58	1	90
	**Rodentia**	**Rat**	1	1	0	24	39	0	65
		**Mouse**	1	1	0	27	130	0	159
**Laurasiatheri**	**Carnivora**	**Dog**	1	0	1	9	4	1	16
		**Cat**	1	2	0	6	20	0	29
	**Perissodactyla**	**Horse**	1	0	1	12	2	1	17
	**Cetartiodactyla**	**Cow**	0	0	0	8	0	1	9
**Xenarthra**		**Armadillo**	1	2	0	9	0	1	13
**Afrotheria**		**Tenrec**	0	0	0	0	0	0	0
		**Elephant**	0	1	0	1	70	1	73

Based on the genomic location information of each snoRNA gene, we illustrated the precise distribution of the imprinted snoRNA genes in the PWS homologous loci of representative species (Figure 1). The size of the PWS imprinted snoRNA cluster was notably expanded in rodents. The cluster sizes of both mouse and rat were approximately two-fold that of the human. In both the rat and mouse genomes, the PWS-related snoRNA clusters were extended by the insertion of a long heterogeneous fragment containing no snoRNA genes. However, the insertion sites were different, occurring within the HBII-52 gene family in the rat genome and in the HBII-85 gene family in mouse. Interestingly, the size of the imprinted snoRNA cluster does not appear to determine the gene number. Despite similar cluster sizes, the dog has only 25% of the snoRNA genes presented in human. These results imply that the PWS imprinted snoRNA cluster is located in a highly active genomic region, a hot spot for chromosomal rearrangements and gene duplications as observed in other imprinted loci [54].

### Phylogenetic Analyses

**Figure 2:**
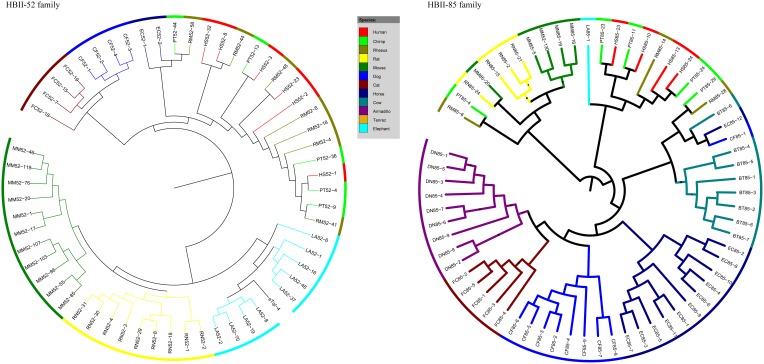
Phylogenetic analysis of HBII-52 and HBII-85 gene family. Abbreviations: HS, human; PT, chimpanzee; RM, rhesus; MM, mouse; RN, rat; CF, dog; FC, cat; EC, horse; BT, cow; DN, armadillo; LA, elephant.

The phylogenetic network indicated that each gene families could form a single phylogenetic clade (Figure. S1). The phylogenetic relationship among the gene families exhibits consistence with their physical distribution in the chromosome. For example, HBII-436 and HBII-13, which are closely distributed in the locus, showed close phylogenetic relationship. Interestingly, the HBII-438 family, consisting of two gene members HBII-438A and HBII-438B, was separated by the large gene cluster of HBII-52 and HBII-85. This suggests these two gene cluster might origin from an insertion of ancestral gene(s) within the HBII-438 family and followed by extensive expansions [33]. Figure 2 shows the intra-family gene relationship of the HBII-52 and the HBII-85 family. A remarkable feature exhibited by the phylogenetic trees is that gene members from same species always form a single clade, especially in non-primates species, suggesting their close relationship. In primates, an intermixed pattern among species was observed, implying of a progressive evolutionary process of these gene families. This pattern was also suggested by the phylogenetic network shown in Figure S1.

### Recent Creation of PWS-related Imprinted snoRNA Genes through a Birth-and-death Process

**Figure 3:**
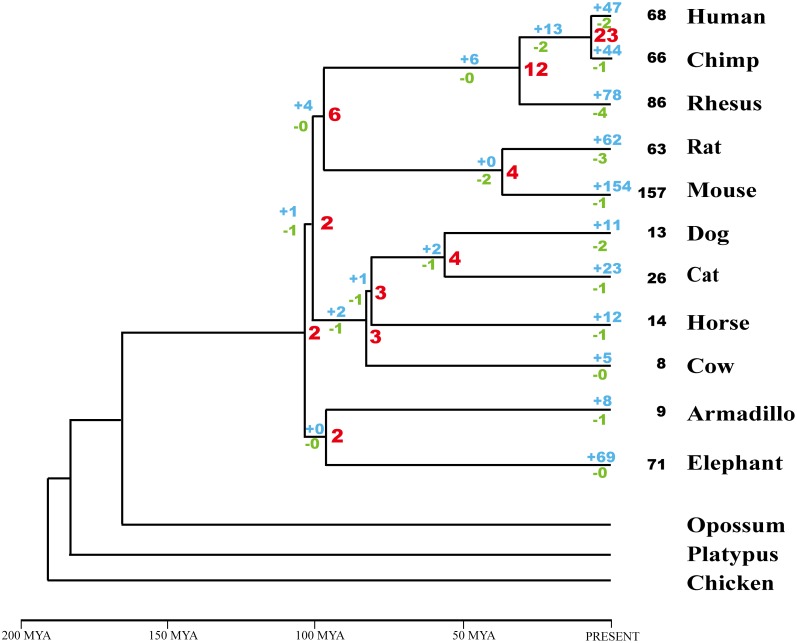
Gene numbers and changes in PWS-related imprinted snoRNA genes with respect to eutherian divergence. Numbers in black indicate the current gene number in each species, whereas red numbers indicate the ancestral gene number, blue numbers denote gained genes, and green numbers indicate lost genes. The major diversification events of eutherians are indicated on the timescale. MYA, a million years ago.

To further explore the evolutionary history of the PWS-related imprinted snoRNA genes, we estimated the number of imprinted snoRNA genes in all ancestral organisms and their increases and decreases at different stages during the evolution of eutherian. Considering that their functional importance and gene volume, we focused this analysis on the two multi-gene families, HBII-52 and HBII-85. To estimate the gene number in ancestral species as well as gene gains and losses, we used the reconciled tree method [42]–[44], in which the topology of a gene tree is reconciled with that of a species tree. The analysis was based on the “Euarchontoglires tree” proposed by Murphy et al. [55], which suggested that primates and rodents are sister groups forming a clade named Euarchontoglires. Figure 3 summarizes the gene numbers estimated for each ancestral species and the associated changes along the different lineages. The numbers of PWS-related imprinted snoRNA genes were extremely low in ancestral organisms as compared with extant species. Interestingly, the evolutionary history of PWS-related snoRNA families is dominated by gene increases (Figure 3). In both the HBII-52 and HBII-85 families (Figure S2 and S3), most (55–98%) of the existing family members arose from lineage-specific gene gain events. In the human genome, 31 of the 41 HBII-52 genes were gained after the species diverged from chimpanzees; for the mouse, 127 of the 130 HBII-52 genes were gained after the species diverged from rats. In contrast, gene loss events were very rare. Similar results were observed for the HBII-85 cluster. Figure 3 provided an overview of the evolutionary dynamics of PWS-related imprinted snoRNA genes in mammals, which reflects the rapid gene turnover in these families. It also suggeststhat the extant genes in each species primarily arose through a lineage-specific gene gaining process occurring in each species’ recent history. This trend can also be intuitively inferred by their grouping patterns, i.e., gene members from the same species were always clustered together (Figure S1 and Figure 2).

Since the results suggest rapid intra-species gene expansion occurred during eutherian evolution, especially in primates and rodents, we want to ask how this happened. For this purpose, combined physical-phylogenetic maps of the HBII-52 and HBII-85 gene families in human and mouse were constructed to investigate gene duplication processes (Figure S4). There are several models proposed for gene duplication [56]. The tandem duplication model predicts a close phylogenetic relationship for physically neighboring genes. This pattern was observed in all the investigated gene families, i.e. HBII-52, HBII-85, MBII-52 and MBII-85. However, in the HBII-52 and MBII-52 family, a lot of physically distant genes show phylogenetic connections, which seems like the duplicated genes have jumped or trans-located from their original sites. This pattern could be explained with a ‘regional ectopic duplication’ model, which usually refers to duplication of individual or a small group of genes to an unlinked locus [57], [58]. The mechanism behind this may be recombination during the gametogenesis. These results also suggest different duplication processes were favored by different gene families. The tandem duplication plays a major role in the recent expansion of the HBII-85 and MBII-85 family; while the regional ectopic duplication dominates the expansion process of the HBII-52 and MBII-52 families.

The birth-and-death model of gene evolution predicts the death of genes, or “gene degradation”. Consistent with this idea, we found pseudogenes in all species (Table S3, Figure S5). Gene degradation mainly occurs through the loss-of-function mutations that impair snoRNA processing or snoRNP formation, e.g., in the C/D boxes. Primates and rodents generally exhibit much lower pseudogene ratios than other species (Table S3). For example, in the HBII-52 family, the average pseudogene ratios were 0.138 and 0.158 for primates and rodents, respectively, but 0.667 for other species. This observations suggest that a trend toward gene expansion was favored in both primates and rodents and explains why these groups have more PWS-related imprinted snoRNA genes.

### Rapid Evolution of PWS-related Imprinted snoRNAs with Functional Constraints

We next asked whether the PWS-related imprinted snoRNAs have undergone rapid evolution. The evolutionary rates of PWS-related imprinted snoRNAs were investigated by two aspects, the first is the birth rate of new genes and the second is sequence substitution rates. Table 2 presents the birth rate of new genes for both the imprinted and non-imprinted snoRNA gene families. Only the C/D box non-imprinted snoRNAs were included in this comparison because all imprinted snoRNAs belong to this class. In the PWS locus, 6.9 and 4.1 new genes were born per million years (Mys) in the human and the mouse respectively, while 8.4 and 2.9 non-imprinted snoRNAs were born per Mys throughout the rest of the genome. Note that the number of non-imprinted gene families involved in this calculation is much larger than that of the imprinted snoRNAs, i.e., ∼180 vs. 2. When the number of gene families was taken into account, the average gene birth rate of the imprinted clusters is over 80-fold faster than that of the non-imprinted snoRNAs. This rate increased to 127 folds in mouse. The higher gene birth rate observed in mouse may account for the emergence of many rodent-specific imprinted snoRNAs. The high gene birth rate was also observed in other small non-coding RNAs, such as microRNA [59], [60]. However, the high birth rate of the imprinted snoRNA genes is quite remarkable, even as compared with the microRNAs. A birth rate of 12 new microRNA genes per Mys has been reported in the fruit fly; however, only 0.3 genes/Mys have been preserved over long stretches of time [61]. In fact, only 5 new, consistently expressed microRNA genes have been maintained in the *Drosophila* genus over the last 55 Mys of evolution [62].

**Table 2:** Birth rates of new genes among PWS-related imprinted and non-imprinted snoRNAs.

	Imprinted snoRNA gene clusters					Non-imprinted snoRNA genes	
	Human	Chimp	Rhesus	Mouse	Rat	Human	Mouse
New genes	47	44	78	154	62	57	108
Rate of birth (per Mys)	6.96	6.52	2.52	4.17	1.68	8.44	2.92
Rate of birth per family (per Mys)	3.48	3.26	1.26	2.085	0.84	0.0433	0.0163

Ratios of gene birth rate between imprinted and non-imprinted snoRNAs: human: 80.37; mouse: 127.91.

**Figure 4:**
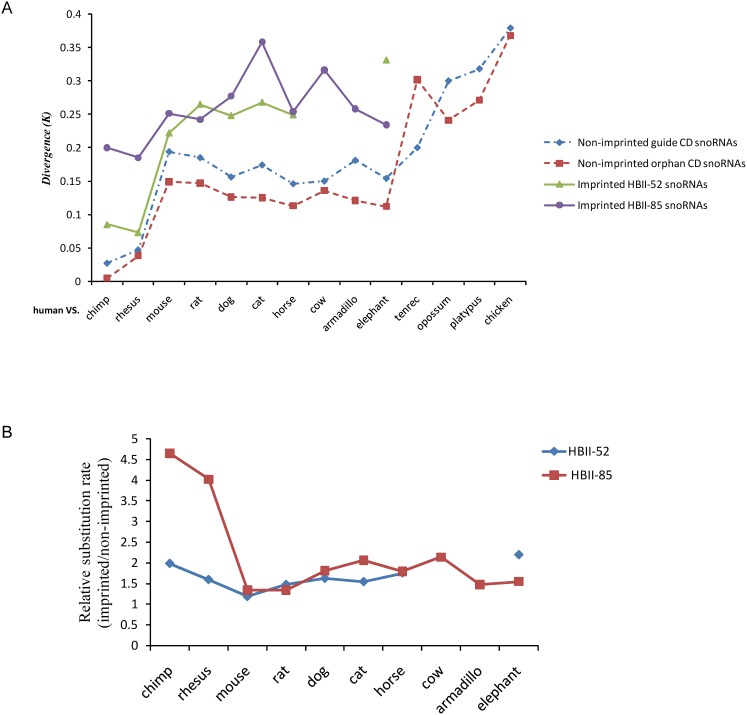
Evolutionary divergence of PWS-related imprinted vs. non-imprinted snoRNA gene families between the human and other mammals. (A) The evolutionary divergence of HBII-52, HBII-85, non-imprinted guide and orphan snoRNA genes between human and other species. *K* denotes the number of substitutions per site. (B) Relative sequence substitution rate between imprinted and non-imprinted snoRNA genes of eutherian mammals.

We also observed rapid sequence evolution of the PWS-related imprinted snoRNAs. Figure 4A shows the evolutionary rates of non-imprinted (including guide and orphan) and the imprinted snoRNA families between human and the other analyzed mammals (the standard errors are indicated in Table S4). This comparison spanned the full range of mammals, and *K* represents the number of substitutions per site. For non-imprinted snoRNAs, the mean *K* value was calculated for all the homologous snoRNA families that are conserved between the human and each of the other species (Table S2); for imprinted snoRNAs, the *K* value was calculated for the HBII-52 and HBII-85 families. This analysis suggests that PWS-related imprinted snoRNA families evolved with a higher sequence substitution rate than non-imprinted snoRNAs. Since orphan snoRNA have no known targeting site on rRNA or snRNAs, it is speculating that they are subjected to relative rapid evolution than guide snoRNAs due to the lack of selective constrains. But our results did not support this notion (Figure 4A). Depending on the species, the HBII-52 family evolved 1.2 to 2.2-fold faster than non-imprinted snoRNAs, with an average value of 1.6 folds; the HBII-85 family evolved 1.3 to 4.6-fold faster than non-imprinted snoRNAs, with an average value of 2.2 folds. Primate species exhibited the highest relative evolution rates (imprinted vs. non-imprinted) of the HBII-85 family (Figure 4B, Table S4). Assuming that the mean evolutionary rate of non-imprinted snoRNAs is nearly constant over a long time scale, this pattern suggests that the evolution of this snoRNA family may have been accelerated in primates.

To determine whether the rapid sequence evolution is specific to the PWS-related imprinted snoRNAs or spans the entire imprinted locus, we compared the substitution rates of the imprinted snoRNA genes and their flanking sequences. A similar comparison was performed using non-imprinted snoRNAs as the reference. We calculated the between-species substitution rates (human vs. chimpanzee and human vs. rhesus monkey) for the snoRNAs (K_sno_) and the flanking genomic sequences (K_f_) (presumably nonfunctional, Table 3). The average K_f_ values were similar between the PWS-related imprinted snoRNAs and the non-imprinted snoRNAs, i.e., 0.011 vs. 0.012 for human vs. chimp and 0.085 vs. 0.078 for human vs. rhesus, indicating nearly equivalent substitution rates. However, the PWS-related imprinted snoRNA cluster had a much higher average K_sno_ as compared with the non-imprinted snoRNAs (0.006 VS. 0.001 and 0.033 VS. 0.013), again supporting the proposed rapid evolution of the PWS-related imprinted snoRNAs. These results suggest that the rapid evolution of PWS-related imprinted snoRNAs was not conferred by a similar process operating in the entire imprinted region but was specific to the snoRNA genes.

**Table 3:** Genetic divergence of snoRNA genes and their flanking sequences in primates for both imprinted and non-imprinted snoRNAs.

	Imprinted region		Non-imprinted region	
	Kf	Ksno	Kf	Ksno
HS-PT	0.011 (0.001)	0.006 (0.002)	0.012 (0.001)	0.001 (0.001)
HS-RM	0.085 (0.002)	0.033 (0.005)	0.078 (0.003)	0.013 (0.004)

Species abbreviation: HS, humans; PT, chimpanzees; RM, rhesus. Standard deviations are shown in parentheses.

**Figure 5:**
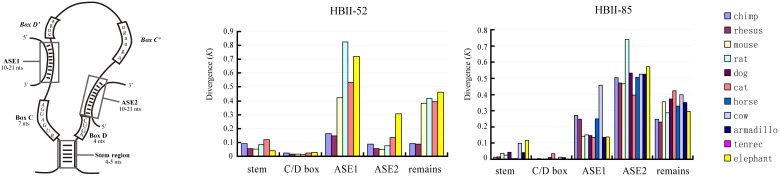
Evolutionary divergence rates for each part of the imprinted snoRNA genes between the human and other mammals. A diagram of box C/D snoRNA structure with different partitions was illustrated in the left [modified from 25]. *K* denotes the number of substitutions per site. Each color represents a comparison between human and another species.

We further investigated the evolutionary patterns of different parts of the imprinted snoRNA genes. As mentioned above, snoRNA could be divided into 5 parts: the stem region, C/D boxes, antisense element 1 (ASE1), antisense element 2 (ASE2) and the remaining sequence. The stem regions, spanning 4–5 nt at each end of a given snoRNA, form a short stem that brings the conserved C and D boxes close to one another to form the structural core motif of the snoRNA. This core motif, referred to as the stem-box structure, coordinates the specific binding of snoRNP proteins and is critical for snoRNA biogenesis and nucleolar localization. The antisense elements are complementary to rRNA or snRNA and determine the site for 2′-O-ribose methylation or for other cellular targets with other functions [1], [25]. Figure 5 shows the distribution of the evolutionary distance, *K*, corresponding to each part of the snoRNA gene between the human and the other analyzed species. The distribution clearly shows that different parts of the genes were subjected to different levels of selection constraint. In general, the C/D boxes and the stem regions were the most conserved, with *K* values under or approximately 0.1, whereas the evolutionary rates of the other three regions varied depending on the gene family. For the HBII-52 family, the two antisense elements showed significant different evolutionary rates, with the ASE1 evolving at an average of ∼4-fold faster than the ASE2 (*p*<0.05, two-tailed t-test). This pattern is consistent with the functional roles of these elements, as the ASE2, but not ASE1, region is reported to form an 18-nt long base pairing with 5-HT_2C_R pre-mRNA to ensure its proper modification [4], [19], [20]. This result suggests that the ASE2 region of HBII-52 has been subjected to strong functional selective constraints. The evolution rate of the remaining sequence was between that of the ASE1 and ASE2, a result that might be partially attributed to the involvement of two putatively functional motifs, namely, the C’ and D’ boxes, in this region [1]. Based on our analysis, the general evolution rate of different parts of the HBII-52 snoRNA genes falls in the order of ASE1>remaining sequence>ASE2>stem region>C/D boxes. For the HBII-85 families, the ASE1 region evolved with a significant lower rate than ASE2 (*p*<0.01, two-tailed t-test) and might have been subjected to functional selection. The molecular target of HBII-85 snoRNAs has yet to be identified; and our results suggest that their potential target(s) are determined by the ASE1 region.

### Positive Selection and Adaptive Signature of PWS-related Imprinted snoRNA Clusters in Euarchontoglires

Tajima’s *D* test is widely used to detect positive selection on non-coding sequences [34], [63]. To exam whether the imprinted snoRNA gene families in Euarchontoglires are subjected to positive selection, the Tajima’s *D* test was conducted on the HBII-52 and the HBII-85 families of each species. The application is based on the consideration that the gene members within a genome are genetically closely related and originate from common ancestors. The Tajima’s *D* values for the HBII-52 family in Euarchontoglires are all negative with remarkable significance (Table 4), suggesting these sequences are subjected to positive selection. Interestingly, although 70 HBII-52 gene members were found in the elephant genome, the Tajima’s *D* value is non-significant (data not shown). The Tajima’s *D* values for HBII-85 family of Euarchontoglires are all negative, but only significant for mice and rats, suggesting a relieved selection force on the primates genomes. For comparison, the 6 adjacent protein-coding genes in the same imprinted locus were also tested for selection. The K_a_/K_s_ values for the six protein-coding genes were all <<1; half of them were suggested to subject to purifying selection (Table 5). Since these protein-coding genes are falling in the same imprinted region as the snoRNA cluster, this result suggests again that the selection is specific for the PWS-related snoRNAs rather than for the entire imprinted region.

**Table 4:** Tajima’s *D* test of Euarchontoglires imprinted snoRNA gene family.

	HBII-52					HBII-85				
	HS	PT	RM	MM	RN	HS	PT	RM	MM	RN
Tajima’s *D*	**−2.07**	**−1.91**	**−2.13**	**−2.48**	**−2.55**	−1.27	−1.16	−1.11	**−2.41**	**−2.48**
*P* value	<0.05	<0.05	<0.05	<0.01	<0.001	>0.10	>0.10	>0.10	<0.01	<0.001

Species abbreviation: HS, humans; PT, chimpanzees; RM, rhesus; MM, mice; RN, rats. The significant (*P*<0.05) test results were labeled bold.

**Table 5:** Ka/Ks tests of protein-coding genes in the PWS locus.

	Protein-coding genes					
Gene	UBE3A	SNRPN	ATP10A	NDN	MKRN3	MAGEL2
Ks	0.004	0	0.024	0.004	0.011	0.033
Ka	0	0	0.004	0	0.002	0.004
Ka/Ks	0	-	0.167	0	0.182	0.121
Purifying selection	0.151	ND	**0**	0.151	**0.045**	**0.029**

*P* values<0.05 for purifying selection are in bold.

**Figure 6:**
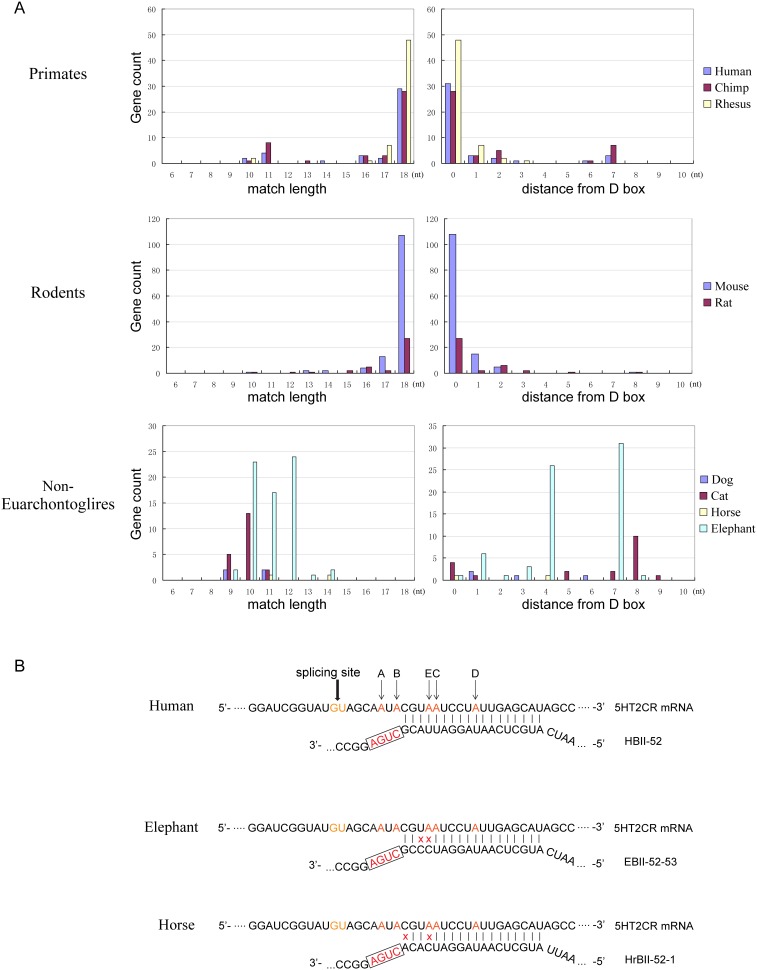
The binding pattern between HBII-52 and 5-HT_2C_R mRNA in mammals. (A) Left panels indicate the length distribution of perfect base-pairing between HBII-52 and 5-HT_2C_R mRNA. Right panels indicate the distance of base-pairing from the D box motif. Analyses of primates, rodents and other mammals are indicated with different colors. In primates and rodents, the length of the base-pairing was centered approximately 18 nt and started just upstream of the D box. In other species, the length of the base-pairing ranged from 9–14 nt and started several nucleotides away from the D box. (B) The best base complementarity between the antisense element of the human, elephant or horse HBII-52 snoRNA and the corresponding 5-HT_2C_R receptor. Nucleotides in red indicate the A to I editing sites (A to E); nucleotides in orange indicate proximal splice site. Box, D box. Red crosses indicate mismatch within the RNA duplex.

The HBII-52 snoRNAs are thought to regulate the modification and/or splicing of 5-HT_2C_R pre-mRNA through an 18-nt long base pairing. Since 5-HT_2C_R is an important neural signal transduction molecule, abnormal regulation of 5-HT_2C_R by HBII-52 was proposed to be involved in the PWS. We therefore investigated whether the regulation of 5-HT_2C_R by HBII-52 is a universal phenomenon in eutherians. For this purpose, the binding pattern between HBII-52 and 5-HT_2C_R mRNA was determined in a number of species (Figure 6A). The results showed that the 18-nt perfect match between HBII-52 and 5-HT_2C_R only presented in primates and rodents. Because the D box of snoRNA is vital for snoRNPs binding, the start site of the pairing was also investigated. Similarly, in primates and rodents, most base pairing began just after the D box, while in other species the start sites were random relative to the D box. To further comparing the HBII-52∶5HT_2C_R binding patterns between primates/rodents and the other mammals, we investigated the base complementarity of the longest continuous perfect matches in humans, elephants and horses respectively (Figure 6B). The data showed that in elephants and horses there are two mismatches within the first five nucleotide positions upstream of the D box, which is important for the efficient modification of the fifth nucleotide, i.e. the C site of the A-I editing of 5HT_2C_R mRNA [64], [65]. These results therefore imply that the regulatory role of HBII-52 might first evolve in the common ancestor of primates and rodents.

## Discussion

### Rapid Evolution of the PWS-related Imprinted snoRNA Clusters via a Birth-and-death Process

SnoRNA genes have traditionally been regarded as conserved components of the genome, mainly due to their function of guiding the methylation and pseudouridylation of rRNA. Most snoRNA genes show great homogeneity among distant species, even between humans and yeast. In striking contrast, the snoRNAs in the imprinted PWS locus have undergone rapid evolution during the diversification of mammals. We observed substantial increases in gene number of the PWS-related imprinted snoRNAs in primates and rodents, and their gene birth rates were more than 80-fold higher than the non-imprinted snoRNAs. At the same time, their sequence evolution rate was also significantly higher than that of the non-imprinted snoRNAs. It is therefore of particular interest to study the evolutionary process underlying the rapid evolution of this imprinted snoRNA gene cluster.

Although rapid evolution of small non-coding RNAs (sncRNAs) has frequently been observed in recent years [59], [60], few reports have investigated the evolutionary history of sncRNA genes in depth [59], [60], [66]. In this study, the remarkable evolutionary dynamics of the HBII-85 and HBII-52 gene families provide a clear example of the birth-and-death evolution. The evidence for this conclusion can be summarized as follows: (i) similar intra- and inter-genomic divergences and close phylogenetic relationships among the intra-species gene members (Table S5, Figure S1); (ii) the functional regions of the imprinted snoRNA genes are subjected to strong selective constraints; and (iii) a number of pseudogenes, representing the remnants of gene death events, were observed pervasively. Moreover, the birth-and-death process can finely explain the high variations in gene number of the PWS-related snoRNA families.

The balance between gene birth and death might have determined the gene number within the cluster in each species. In primates and rodents, the balance tends toward gene birth, while in other lineages, it leans toward gene death. The birth-and-death evolutionary model can also explain the lack of genes in the ancestral species. For such ancestral genes likely to turn to death during the diversification of mammals and were replaced by the newborn lineage-specific genes, which share few common feature among species. Thus, the ancestral gene in the common ancestor of Armadillo/Elephant and the most recent common ancestor of all placental mammals are low.

The rapid evolution of the imprinted snoRNA genes might partially due to the different way they expressed. In contrast to biallelically-expressed genes, imprinted genes are expressed from only one allele with the other allele is repressed. Because of this lack of a backup copy, similar selective force is predicted to trigger more efficient responses from imprinted genes relative to the non-imprinted counterparts. Moreover, concerning the parental interests conflict of imprinted genes, other selective constraints might be evoked, e.g. mother-infant co-adaptation [67], [68]. We therefore anticipate that other imprinted genes will exhibit rapid evolution under certain selective forces. The birth-and-death evolution of multi-gene families has played important roles during the origin and development of some complex genetic systems, such as the adaptive immune system, the homeobox super gene family and the olfactory system [69]. The PWS-related imprinted snoRNA genes have undergone a similar process at an outstanding evolutionary rate. We anticipate that they might be involved in the development of a complex system that has elaborately evolved in the mammalian lineage.

### The Evolutionary Pattern of Other Imprinted Small Non-coding RNAs

**Figure 7:**
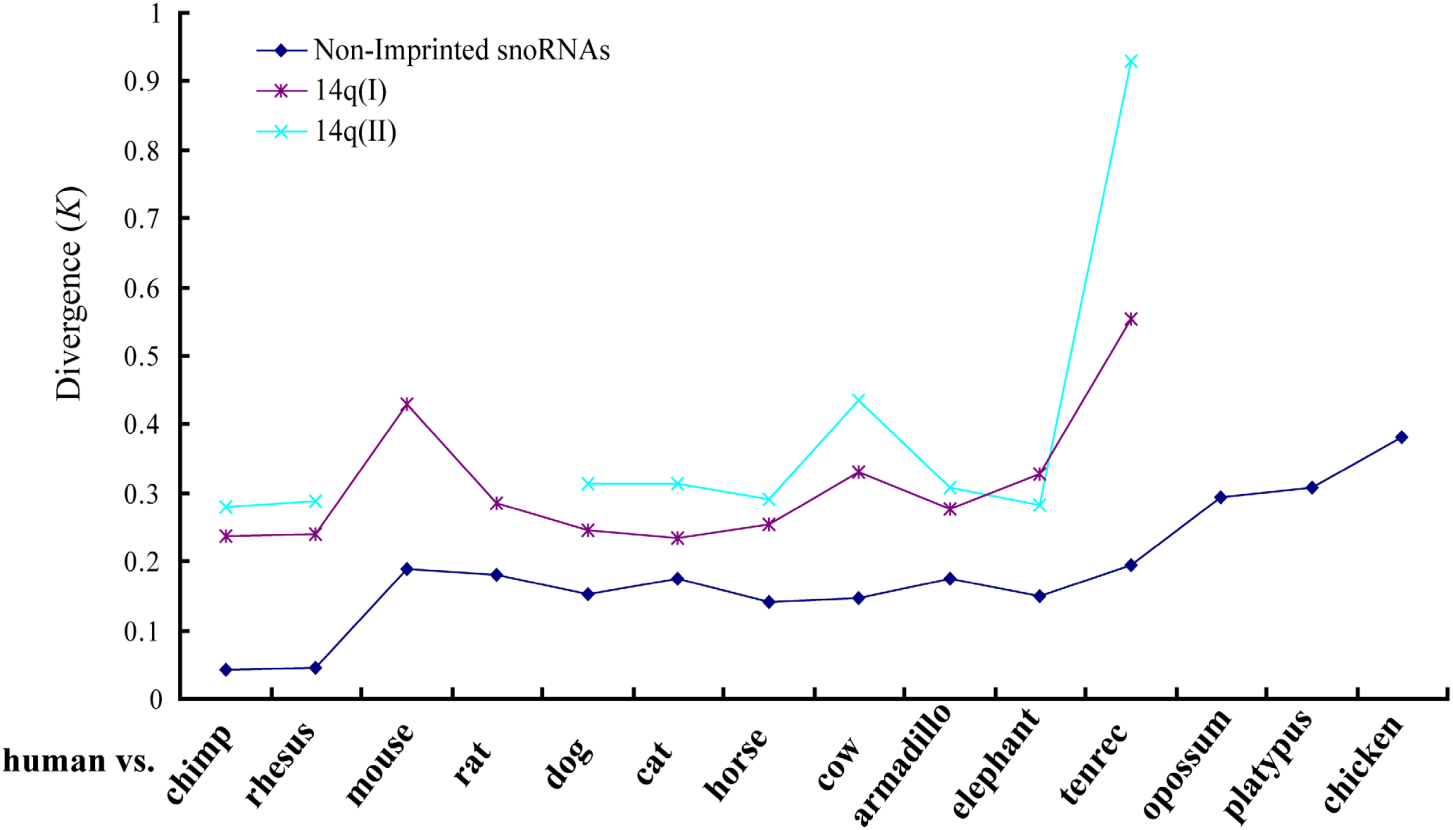
Evolutionary divergence of imprinted 14q(I) and 14q(II) snoRNA family vs. non-imprinted snoRNA gene families between the human and other mammals. *K* denotes the number of substitutions per site.

**Figure 8:**
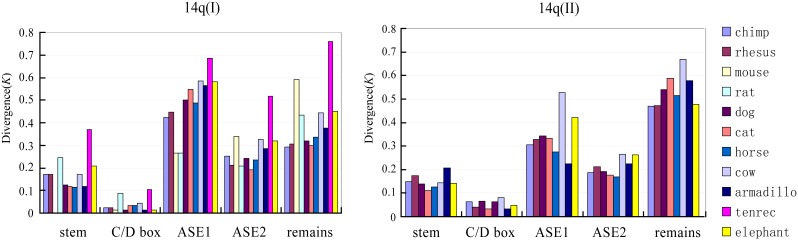
Evolutionary divergence rates for each part of the imprinted snoRNA genes in 14q32 region between the human and other mammals.

Mammals have another large imprinted snoRNA cluster located in the 14q32 region of the human genome. This cluster mainly contain two multi-gene families, the 14qI and 14qII family; a rat-specific imprinted snoRNA gene family, RBII-36, is also located in this region.[4]. Our analyses revealed that the 14qI and 14qII gene family also exhibit rapid sequence substitution than non-imprinted snoRNAs (Figure 7). But significant selective constraint on the ASE was not observed (Figure 8), suggesting a lack of functional constraint on these genes. This finding is consistent with the fact that the function(s) of 14qI or 14qII families is still unknown. The phylogenetic analysis of 14qI family showed a different pattern comparing to that of the HBII-52 or HBII-85 families (Figure S6). The 14qI snoRNA genes were clustered with their orthologous, a sign of divergent evolution. Thus, our results indicate that imprinted snoRNA genes located in different genome regions are subjected to different evolution process, which might be highly related to the selective force they received. Further determining the biological functions of the imprinted snoRNA genes will help to unveil the significance of their rapid evolution.

There are studies suggest positive selection for microRNAs [70]. The microRNA cluster located in the *DLK-DIO3* imprinted locus is the largest microRNA cluster discovered thus far. A study on the evolution of this microRNA cluster revealed that most of the experimentally validated miRNAs could be detected in nearly all the sequenced eutherian genomes [71]. This finding suggests that the structure of this miRNA cluster is evolutionarily stable, which is different from the evolutionary pattern of the PWS-related snoRNA cluster in this study.

### Adaptive Evolution of the PWS-related snoRNA Gene Cluster and Neural Development in Euarchontoglires

The PWS-related imprinted snoRNAs in Euarchontoglires exhibit adaptive signatures. A population data based study on the HBII-52 gene family of human suggested that positive selection and gene conversion have driven the evolution of the imprinted snoRNA gene cluster [34]. Consistent with this report, our study also detected remarkable positive selection on the HBII-52 gene family as well as the HBII-85 gene family in primates and rodents. In addition, primates and rodents have more imprinted snoRNA genes than other placental mammals (with the exception of elephants). Changes in gene number often are accompanied by the adaptation of a species to its environment. For example, an analysis of the changes in olfactory receptor gene number among mammals revealed that primates and the platypus have fewer genes than other mammals [45]. The development of the trichromatic vision system in primates greatly improved their visual sensory abilities, thereby reducing the importance of olfactory sensation. The platypus, in turn, has adapted to a semi-aquatic lifestyle and relies less heavily on olfactory sensation. Similarly, the remarkable increase in PWS-related imprinted snoRNA genes might be also needed to meet certain functional demands during mammalian evolution.

Three lines of evidence suggest that the PWS-related imprinted snoRNAs are undergoing functional evolution: (i) the imprinted snoRNAs have different expression patterns among mammals, e.g. human HBII-85 snoRNAs have a wider expression profile than MBII-85 and RBII-85 (MBII-85 and RBII-85 are the homolog of HBII-85 in mice and rats, respectively)[7], which might closely relate to their functional diversification; (ii) at the phenotypic level, the site-specific deletion of the entire murine MBII-85 gene cluster did not result in obesity, a major phenotype observed in the human PWS [24]; (iii) the interactions between the HBII-52 snoRNAs and its target, 5-HT_2C_R mRNA, are different among mammalian lineages. Our results showed that the 18-nt perfect match between HBII-52 and 5-HT_2C_R was only found in primates and rodents; while in other lineages, the lengths of the continuous perfect match were limited to 14 nts and with mismatches within the five nucleotide upstream box D, which region is important for the precise modification of the fifth nucleotide, i.e. the C site for the A-I editing of 5HT2CR mRNA[19]. Cavaillé and Bachellerie has indicated that two nucleotides of mismatch in the box D-proximal portion will greatly impair the modification efficiency of the target site, even in a long G/C-rich duplex [65]. While, the RNA duplex between HBII-52 and 5HT_2C_R mRNA is A/U rich (11/18), whose interaction is expected to be more sensitive to mismatches and a single mutation could lead to a 100-fold inhibition of the modification in a A/U rich duplex [64]. Thus, the snoRNA guided modifications is most likely invalid here. Moreover, previous studies have demonstrated that mismatches between snoRNA and exon Vb significantly influence the regulation of alternative splicing of 5-HT_2C_R pre-mRNA [20]. Thus, we speculate that the mRNA regulatory function for HBII-52 first evolved in the common ancestor of primates and rodents. Although 70 HBII-52 gene members are found in the elephant genome, they do not have a long complementary sequence to the 5-HT_2C_R pre-mRNA. It is possible that the rapid expansion of the HBII-52 gene family in the elephant genome is for other function(s).

How and to what extent the rapid evolution of PWS-related imprinted snoRNA cluster has contributed to the adaptive evolution of primates and rodents remain unclear. Many imprinted genes play important roles in brain development and functions [9]. Typically, maternally expressed imprinted genes have effects on ‘higher’ cognitive systems, whereas paternal genes have effects on brain systems that fill ‘emotional’ or autonomic functions [72]. The 15q11–13 imprinted snoRNA cluster is paternally expressed in the adult brain [73], suggesting that these genes might be involved in emotional function. Several lines of evidence suggest that the evolution of the 15q11–13 snoRNA cluster has contributed to the fine control of emotion and the related social behaviors of individuals. The HBII-52 snoRNAs could regulatethe 5-HT_2_C receptor pre-mRNA the A-to-I RNA editing and alternative splicing of the 5-HT_2_C receptor pre-mRNA. 5-HT (5-hydroxytryptamine, or serotonin) is a very important monoamine neurotransmitter that is well-known to contribute to feelings of well-being and happiness. The 5-HT_2_C receptor is a subtype of the 5-HT receptor, which regulates mood, anxiety, feeding, and reproductive behavior [74]. HBII-52 can inhibit its RNA editing at the C-site in the nucleolus, which is crucial for decreasing the efficiency for activating the G-proteins. The functionally compromised 5-HT_2_C receptor that results from this mis-editing has been detected in the depressed suicide victims [75]. HBII-52 also participates in the regulation of alternative splicing of the 5-HT_2_C receptor pre-mRNA by promoting exon Vb inclusion. Without exon Vb, the mRNA does not encode a functional 5-HT_2_CR receptor [20]. The evolving HBII-52 snoRNAs may therefore have participated in the fine regulation of the 5-HT neurotransmission pathway by modulating the 5-HT_2_C receptor in primates and rodents.

Emotion is a vital factor affecting individual social behaviors. Two key characteristics of Angelman syndrome are an unusually sociable disposition and reduced display of negative-affect signals (stubbornness and temper tantrums) [13]. Conversely, individuals with PWS show increased negative-affect signals and are prone to mood instability and non-psychotic depression [76], [77]. These findings have led to the idea that, in addition to influencing the resource allocation from the mother to the offspring through effects on, such as, suckling behavior, brain-expressed imprinted genes can also influence the social resources that the offspring receives, especially in the social animals like primates. These resources include food, protection and reproductive opportunities.

As imprinted genes play important roles in brain development and function, it has been suggested these genes might be subject to selection [9]. In this study, we revealed the rapid adaptive evolution of imprinted snoRNA genes in the PWS locus. The accelerated evolution of PWS-related imprinted snoRNA genes in Euarchontoglires might have contributed to the fine-tuning of emotion control and social behavior, which may have provided benefits such as greater allocation of social resources like reproductive opportunities. These consequences are clearly advantageous for the survival of individuals in the animal societies.

## Supporting Information

Figure S1 **Phylogenetic network of imprinted snoRNAs in the PWS locus.** Gene families were indicted with different colors. Species abbreviations: H, human; MM, mouse; RN, rat; CF, dog; BT, cow; DN, armadillo; LA, elephant.
(TIF)

Figure S2 **Gene numbers and changes in in the HBII-52 gene family.** Numbers in black indicate the current gene number in each species, whereas red numbers indicate the ancestral gene number, blue numbers denote gained genes, and green numbers indicate lost genes.
(TIF)

Figure S3 **Gene numbers and changes in in the HBII-85 gene family.**
(TIF)

Figure S4 **Combined physical-phylogenetic map.** Imprinted snoRNA genes are depicted as vertical bar as in figure 1. The consistency between phylogenetic relationship and physical distribution suggest the genes are created by tandem duplication.
(TIF)

Figure S5 **Human HBII-85 and HBII-52 pseudogenes.** Selected pseudogenes were aligned with corresponding normal genes. Red arrow indicate loss-of-function mutations in these pseudo genes.
(TIF)

Figure S6 **Phylogenetic analysis of 14q(I) gene family.** Abbreviations: HS, human; PT, chimpanzee; RM, rhesus; MM, mouse; RN, rat; CF, dog; FC, cat; EC, horse; BT, cow; DN, armadillo; LA, elephant.
(TIF)

Table S1 **Chromosome loci, size, and the genomic position of PWS imprinted regions in 12 eutherians.**
(PDF)

Table S2 **Pseudogenes identified in the PWS imprinted region.**
(PDF)

Table S3 **Conserved non-imprinted box C/D snoRNA gene families between human and other species.**
(PDF)

Table S4 **Nucleotide divergence between human and other species for non-imprinted and imprinted** **snoRNA genes.**
(PDF)

Table S5 **Intra- and inter- genomic divergence for PWS-related imprinted snoRNA genes.**
(PDF)

Data S1 **Sequences of imprinted and non-imprinted snoRNA genes.**
(ZIP)

Data S2 **The sequence alignments for phylogenetic analyses.**
(ZIP)

## References

[1] Bachellerie JP, Cavaille J, Huttenhofer A (2002) The expanding snoRNA world. Biochimie 84: 775–790. 12457565 10.1016/s0300-9084(02)01402-5

[2] Brown JW, Echeverria M, Qu LH (2003) Plant snoRNAs: functional evolution and new modes of gene expression. Trends Plant Sci 8: 42–49. 12523999 10.1016/s1360-1385(02)00007-9

[3] Runte M, Huttenhofer A, Gross S, Kiefmann M, Horsthemke B, et al (2001) The IC-SNURF-SNRPN transcript serves as a host for multiple small nucleolar RNA species and as an antisense RNA for UBE3A. Hum Mol Genet 10: 2687–2700. 11726556 10.1093/hmg/10.23.2687

[4] Cavaille J, Seitz H, Paulsen M, Ferguson-Smith AC, Bachellerie JP (2002) Identification of tandemly-repeated C/D snoRNA genes at the imprinted human 14q32 domain reminiscent of those at the Prader-Willi/Angelman syndrome region. Hum Mol Genet 11: 1527–1538. 12045206 10.1093/hmg/11.13.1527

[5] Cavaille J, Vitali P, Basyuk E, Huttenhofer A, Bachellerie JP (2001) A novel brain-specific box C/D small nucleolar RNA processed from tandemly repeated introns of a noncoding RNA gene in rats. J Biol Chem 276: 26374–26383. 11346658 10.1074/jbc.M103544200

[6] de los Santos T, Schweizer J, Rees CA, Francke U (2000) Small evolutionarily conserved RNA, resembling C/D box small nucleolar RNA, is transcribed from PWCR1, a novel imprinted gene in the Prader-Willi deletion region, which Is highly expressed in brain. Am J Hum Genet 67: 1067–1082. 11007541 10.1086/303106 PMC1288549

[7] Cavaille J, Buiting K, Kiefmann M, Lalande M, Brannan CI, et al (2000) Identification of brain-specific and imprinted small nucleolar RNA genes exhibiting an unusual genomic organization. Proc Natl Acad Sci U S A 97: 14311–14316. 11106375 10.1073/pnas.250426397 PMC18915

[8] Bortolin-Cavaille ML, Cavaille J (2012) The SNORD115 (H/MBII-52) and SNORD116 (H/MBII-85) gene clusters at the imprinted Prader-Willi locus generate canonical box C/D snoRNAs. Nucleic Acids Res 40: 6800–6807. 22495932 10.1093/nar/gks321 PMC3413130

[9] Wilkinson LS, Davies W, Isles AR (2007) Genomic imprinting effects on brain development and function. Nat Rev Neurosci 8: 832–843. 17925812 10.1038/nrn2235

[10] Castle JC, Armour CD, Lower M, Haynor D, Biery M, et al (2010) Digital genome-wide ncRNA expression, including SnoRNAs, across 11 human tissues using polyA-neutral amplification. PLoS One 5: e11779. 20668672 10.1371/journal.pone.0011779 PMC2909899

[11] Peters J (2008) Prader-Willi and snoRNAs. Nat Genet 40: 688–689. 18509309 10.1038/ng0608-688

[12] Belmonte MK, Cook EH Jr, Anderson GM, Rubenstein JL, Greenough WT, et al (2004) Autism as a disorder of neural information processing: directions for research and targets for therapy. Mol Psychiatry 9: 646–663. 15037868 10.1038/sj.mp.4001499

[13] Bolton PF, Veltman MW, Weisblatt E, Holmes JR, Thomas NS, et al (2004) Chromosome 15q11–13 abnormalities and other medical conditions in individuals with autism spectrum disorders. Psychiatr Genet 14: 131–137. 15318025 10.1097/00041444-200409000-00002

[14] Cook EH Jr, Scherer SW (2008) Copy-number variations associated with neuropsychiatric conditions. Nature 455: 919–923. 18923514 10.1038/nature07458

[15] de Smith AJ, Purmann C, Walters RG, Ellis RJ, Holder SE, et al (2009) A deletion of the HBII-85 class of small nucleolar RNAs (snoRNAs) is associated with hyperphagia, obesity and hypogonadism. Hum Mol Genet 18: 3257–3265. 19498035 10.1093/hmg/ddp263 PMC2722987

[16] Sahoo T, del Gaudio D, German JR, Shinawi M, Peters SU, et al (2008) Prader-Willi phenotype caused by paternal deficiency for the HBII-85 C/D box small nucleolar RNA cluster. Nat Genet 40: 719–721. 18500341 10.1038/ng.158 PMC2705197

[17] Duker AL, Ballif BC, Bawle EV, Person RE, Mahadevan S, et al (2010) Paternally inherited microdeletion at 15q11.2 confirms a significant role for the SNORD116 C/D box snoRNA cluster in Prader-Willi syndrome. Eur J Hum Genet 18: 1196–1201. 20588305 10.1038/ejhg.2010.102 PMC2987474

[18] Runte M, Varon R, Horn D, Horsthemke B, Buiting K (2005) Exclusion of the C/D box snoRNA gene cluster HBII-52 from a major role in Prader-Willi syndrome. Hum Genet 116: 228–230. 15565282 10.1007/s00439-004-1219-2

[19] Vitali P, Basyuk E, Le Meur E, Bertrand E, Muscatelli F, et al (2005) ADAR2-mediated editing of RNA substrates in the nucleolus is inhibited by C/D small nucleolar RNAs. J Cell Biol 169: 745–753. 15939761 10.1083/jcb.200411129 PMC2171610

[20] Kishore S, Stamm S (2006) The snoRNA HBII-52 regulates alternative splicing of the serotonin receptor 2C. Science 311: 230–232. 16357227 10.1126/science.1118265

[21] Kishore S, Khanna A, Zhang Z, Hui J, Balwierz PJ, et al (2010) The snoRNA MBII-52 (SNORD 115) is processed into smaller RNAs and regulates alternative splicing. Hum Mol Genet 19: 1153–1164. 20053671 10.1093/hmg/ddp585 PMC2838533

[22] Doe CM, Relkovic D, Garfield AS, Dalley JW, Theobald DE, et al (2009) Loss of the imprinted snoRNA mbii-52 leads to increased 5htr2c pre-RNA editing and altered 5HT2CR-mediated behaviour. Hum Mol Genet 18: 2140–2148. 19304781 10.1093/hmg/ddp137 PMC2685753

[23] Ding F, Li HH, Zhang S, Solomon NM, Camper SA, et al (2008) SnoRNA Snord116 (Pwcr1/MBII-85) deletion causes growth deficiency and hyperphagia in mice. PLoS ONE 3: e1709. 18320030 10.1371/journal.pone.0001709 PMC2248623

[24] Skryabin BV, Gubar LV, Seeger B, Pfeiffer J, Handel S, et al (2007) Deletion of the MBII-85 snoRNA gene cluster in mice results in postnatal growth retardation. PLoS Genet 3: e235. 18166085 10.1371/journal.pgen.0030235 PMC2323313

[25] Kiss T (2002) Small nucleolar RNAs: an abundant group of noncoding RNAs with diverse cellular functions. Cell 109: 145–148. 12007400 10.1016/s0092-8674(02)00718-3

[26] Piekna-Przybylska D, Decatur WA, Fournier MJ (2007) New bioinformatic tools for analysis of nucleotide modifications in eukaryotic rRNA. Rna 13: 305–312. 17283215 10.1261/rna.373107 PMC1800513

[27] Lestrade L, Weber MJ (2006) snoRNA-LBME-db, a comprehensive database of human H/ACA and C/D box snoRNAs. Nucleic Acids Res 34: D158–162. 16381836 10.1093/nar/gkj002 PMC1347365

[28] Schmitz J, Zemann A, Churakov G, Kuhl H, Grutzner F, et al (2008) Retroposed SNOfall–a mammalian-wide comparison of platypus snoRNAs. Genome Res 18: 1005–1010. 18463303 10.1101/gr.7177908 PMC2413151

[29] Edwards CA, Mungall AJ, Matthews L, Ryder E, Gray DJ, et al (2008) The evolution of the DLK1-DIO3 imprinted domain in mammals. PLoS Biol 6: e135. 18532878 10.1371/journal.pbio.0060135 PMC2408620

[30] Rapkins RW, Hore T, Smithwick M, Ager E, Pask AJ, et al (2006) Recent assembly of an imprinted domain from non-imprinted components. PLoS Genet 2: e182. 17069464 10.1371/journal.pgen.0020182 PMC1626109

[31] Zhang Y, Qu L (2009) Non-coding RNAs and the acquisition of genomic imprinting in mammals. Sci China C Life Sci 52: 195–204. 19294344 10.1007/s11427-009-0035-2

[32] Royo H, Cavaille J (2008) Non-coding RNAs in imprinted gene clusters. Biol Cell 100: 149–166. 18271756 10.1042/BC20070126

[33] Nahkuri S, Taft RJ, Korbie DJ, Mattick JS (2008) Molecular evolution of the HBII-52 snoRNA cluster. J Mol Biol 381: 810–815. 18616950 10.1016/j.jmb.2008.06.057

[34] Ogorelkova M, Navarro A, Vivarelli F, Ramirez-Soriano A, Estivill X (2009) Positive selection and gene conversion drive the evolution of a brain-expressed snoRNAs cluster. Mol Biol Evol 26: 2563–2571. 19651851 10.1093/molbev/msp173

[35] Yang JH, Zhang XC, Huang ZP, Zhou H, Huang MB, et al (2006) snoSeeker: an advanced computational package for screening of guide and orphan snoRNA genes in the human genome. Nucleic Acids Res 34: 5112–5123. 16990247 10.1093/nar/gkl672 PMC1636440

[36] Ronquist F, Huelsenbeck JP (2003) MrBayes 3: Bayesian phylogenetic inference under mixed models. Bioinformatics 19: 1572–1574. 12912839 10.1093/bioinformatics/btg180

[37] Kumar S, Tamura K, Nei M (2004) MEGA3: Integrated software for Molecular Evolutionary Genetics Analysis and sequence alignment. Brief Bioinform 5: 150–163. 15260895 10.1093/bib/5.2.150

[38] Thompson JD, Gibson TJ, Plewniak F, Jeanmougin F, Higgins DG (1997) The CLUSTAL_X windows interface: flexible strategies for multiple sequence alignment aided by quality analysis tools. Nucleic Acids Res 25: 4876–4882. 9396791 10.1093/nar/25.24.4876 PMC147148

[39] Huson DH, Bryant D (2006) Application of phylogenetic networks in evolutionary studies. Mol Biol Evol 23: 254–267. 16221896 10.1093/molbev/msj030

[40] Posada D, Crandall KA (1998) MODELTEST: testing the model of DNA substitution. Bioinformatics 14: 817–818. 9918953 10.1093/bioinformatics/14.9.817

[41] Letunic I, Bork P (2007) Interactive Tree Of Life (iTOL): an online tool for phylogenetic tree display and annotation. Bioinformatics 23: 127–128. 17050570 10.1093/bioinformatics/btl529

[42] Goodman M, Czelusniak J, Moore G, Romero-Herrera AE, Matsuda G (1979) Fitting the gene lineage into its species lineage, a parsimony strategy illustrated by cladograms constructed from globin sequences. Syst Zool 28: 132–168.

[43] Page RD, Charleston MA (1997) From gene to organismal phylogeny: reconciled trees and the gene tree/species tree problem. Mol Phylogenet Evol 7: 231–240. 9126565 10.1006/mpev.1996.0390

[44] Nam J, Nei M (2005) Evolutionary change of the numbers of homeobox genes in bilateral animals. Mol Biol Evol 22: 2386–2394. 16079247 10.1093/molbev/msi229 PMC1464090

[45] Niimura Y, Nei M (2003) Evolution of olfactory receptor genes in the human genome. Proc Natl Acad Sci U S A 100: 12235–12240. 14507991 10.1073/pnas.1635157100 PMC218742

[46] Niimura Y, Nei M (2007) Extensive gains and losses of olfactory receptor genes in Mammalian evolution. PLoS ONE 2: e708. 17684554 10.1371/journal.pone.0000708 PMC1933591

[47] Hedges SB, Dudley J, Kumar S (2006) TimeTree: a public knowledge-base of divergence times among organisms. Bioinformatics 22: 2971–2972. 17021158 10.1093/bioinformatics/btl505

[48] Kimura M (1980) A simple method for estimating evolutionary rates of base substitutions through comparative studies of nucleotide sequences. J Mol Evol 16: 111–120. 7463489 10.1007/BF01731581

[49] Librado P, Rozas J (2009) DnaSP v5: a software for comprehensive analysis of DNA polymorphism data. Bioinformatics 25: 1451–1452. 19346325 10.1093/bioinformatics/btp187

[50] Tajima F (1989) Statistical method for testing the neutral mutation hypothesis by DNA polymorphism. Genetics 123: 585–595. 2513255 10.1093/genetics/123.3.585 PMC1203831

[51] Koonin EV, Wolf YI (2010) Constraints and plasticity in genome and molecular-phenome evolution. Nat Rev Genet 11: 487–498. 20548290 10.1038/nrg2810 PMC3273317

[52] Tamura K, Dudley J, Nei M, Kumar S (2007) MEGA4: Molecular Evolutionary Genetics Analysis (MEGA) software version 4.0. Mol Biol Evol 24: 1596–1599. 17488738 10.1093/molbev/msm092

[53] Royo H, Basyuk E, Marty V, Marques M, Bertrand E, et al (2007) Bsr, a nuclear-retained RNA with monoallelic expression. Mol Biol Cell 18: 2817–2827. 17507654 10.1091/mbc.E06-10-0920 PMC1949380

[54] Sandovici I, Kassovska-Bratinova S, Vaughan JE, Stewart R, Leppert M, et al (2006) Human imprinted chromosomal regions are historical hot-spots of recombination. PLoS Genet 2: e101. 16839189 10.1371/journal.pgen.0020101 PMC1487178

[55] Murphy WJ, Eizirik E, O’Brien SJ, Madsen O, Scally M, et al (2001) Resolution of the early placental mammal radiation using Bayesian phylogenetics. Science 294: 2348–2351. 11743200 10.1126/science.1067179

[56] Leister D (2004) Tandem and segmental gene duplication and recombination in the evolution of plant disease resistance gene. Trends Genet 20: 116–122. 15049302 10.1016/j.tig.2004.01.007

[57] Richly E, Kurth J, Leister D (2002) Mode of amplification and reorganization of resistance genes during recent Arabidopsis thaliana evolution. Mol Biol Evol 19: 76–84. 11752192 10.1093/oxfordjournals.molbev.a003984

[58] Meyers BC, Kozik A, Griego A, Kuang H, Michelmore RW (2003) Genome-wide analysis of NBS-LRR-encoding genes in Arabidopsis. Plant Cell 15: 809–834. 12671079 10.1105/tpc.009308 PMC152331

[59] Meunier J, Lemoine F, Soumillon M, Liechti A, Weier M, et al (2013) Birth and expression evolution of mammalian microRNA genes. Genome Res 23: 34–45. 23034410 10.1101/gr.140269.112 PMC3530682

[60] Zheng GX, Ravi A, Gould GM, Burge CB, Sharp PA (2011) Genome-wide impact of a recently expanded microRNA cluster in mouse. Proc Natl Acad Sci U S A 108: 15804–15809. 21911408 10.1073/pnas.1112772108 PMC3179086

[61] Lu J, Shen Y, Wu Q, Kumar S, He B, et al (2008) The birth and death of microRNA genes in Drosophila. Nat Genet 40: 351–355. 18278047 10.1038/ng.73

[62] Lu J, Fu Y, Kumar S, Shen Y, Zeng K, et al (2008) Adaptive evolution of newly emerged micro-RNA genes in Drosophila. Mol Biol Evol 25: 929–938. 18296702 10.1093/molbev/msn040 PMC3707409

[63] Wang Y, Shen D, Bo S, Chen H, Zheng J, et al (2010) Sequence variation and selection of small RNAs in domesticated rice. BMC Evol Biol 10: 119. 20429951 10.1186/1471-2148-10-119 PMC2887405

[64] Cavaille J, Nicoloso M, Bachellerie JP (1996) Targeted ribose methylation of RNA in vivo directed by tailored antisense RNA guides. Nature 383: 732–735. 8878486 10.1038/383732a0

[65] Cavaille J, Bachellerie JP (1998) SnoRNA-guided ribose methylation of rRNA: structural features of the guide RNA duplex influencing the extent of the reaction. Nucleic Acids Res 26: 1576–1587. 9512526 10.1093/nar/26.7.1576 PMC147472

[66] Zhang R, Wang YQ, Su B (2008) Molecular evolution of a primate-specific microRNA family. Mol Biol Evol 25: 1493–1502. 18417486 10.1093/molbev/msn094

[67] Plagge A, Gordon E, Dean W, Boiani R, Cinti S, et al (2004) The imprinted signaling protein XL alpha s is required for postnatal adaptation to feeding. Nat Genet 36: 818–826. 15273686 10.1038/ng1397

[68] Curley JP, Barton S, Surani A, Keverne EB (2004) Coadaptation in mother and infant regulated by a paternally expressed imprinted gene. Proc Biol Sci 271: 1303–1309. 15306355 10.1098/rspb.2004.2725 PMC1691726

[69] Nei M, Rooney AP (2005) Concerted and birth-and-death evolution of multigene families. Annu Rev Genet 39: 121–152. 16285855 10.1146/annurev.genet.39.073003.112240 PMC1464479

[70] Li J, Liu Y, Xin X, Kim TS, Cabeza EA, et al (2012) Evidence for positive selection on a number of MicroRNA regulatory interactions during recent human evolution. PLoS Genet 8: e1002578. 22457636 10.1371/journal.pgen.1002578 PMC3310733

[71] Glazov EA, McWilliam S, Barris WC, Dalrymple BP (2008) Origin, evolution, and biological role of miRNA cluster in DLK-DIO3 genomic region in placental mammals. Mol Biol Evol 25: 939–948. 18281269 10.1093/molbev/msn045

[72] Keverne EB, Martel FL, Nevison CM (1996) Primate brain evolution: genetic and functional considerations. Proc Biol Sci 263: 689–696. 8763791 10.1098/rspb.1996.0103

[73] Runte M, Kroisel PM, Gillessen-Kaesbach G, Varon R, Horn D, et al (2004) SNURF-SNRPN and UBE3A transcript levels in patients with Angelman syndrome. Hum Genet 114: 553–561. 15014980 10.1007/s00439-004-1104-z

[74] Heisler LK, Zhou L, Bajwa P, Hsu J, Tecott LH (2007) Serotonin 5-HT(2C) receptors regulate anxiety-like behavior. Genes Brain Behav 6: 491–496. 17451451 10.1111/j.1601-183X.2007.00316.x

[75] Gurevich I, Englander MT, Adlersberg M, Siegal NB, Schmauss C (2002) Modulation of serotonin 2C receptor editing by sustained changes in serotonergic neurotransmission. J Neurosci 22: 10529–10532. 12486144 10.1523/JNEUROSCI.22-24-10529.2002 PMC6758441

[76] Soni S, Whittington J, Holland AJ, Webb T, Maina E, et al (2007) The course and outcome of psychiatric illness in people with Prader-Willi syndrome: implications for management and treatment. J Intellect Disabil Res 51: 32–42. 17181601 10.1111/j.1365-2788.2006.00895.x

[77] Cassidy SB (1997) Prader-Willi syndrome. J Med Genet 34: 917–923. 9391886 10.1136/jmg.34.11.917 PMC1051120

