# Supplementary figures and images for: Rapid Birth-and-Death Evolution of Imprinted snoRNAs in the Prader-Willi Syndrome Locus: Implications for Neural Development in Euarchontoglires
Source: PLoS One. 2014 Jun 19;9(6):e100329. doi: 10.1371/journal.pone.0100329 (PMC4063771; doi:10.1371/journal.pone.0100329)

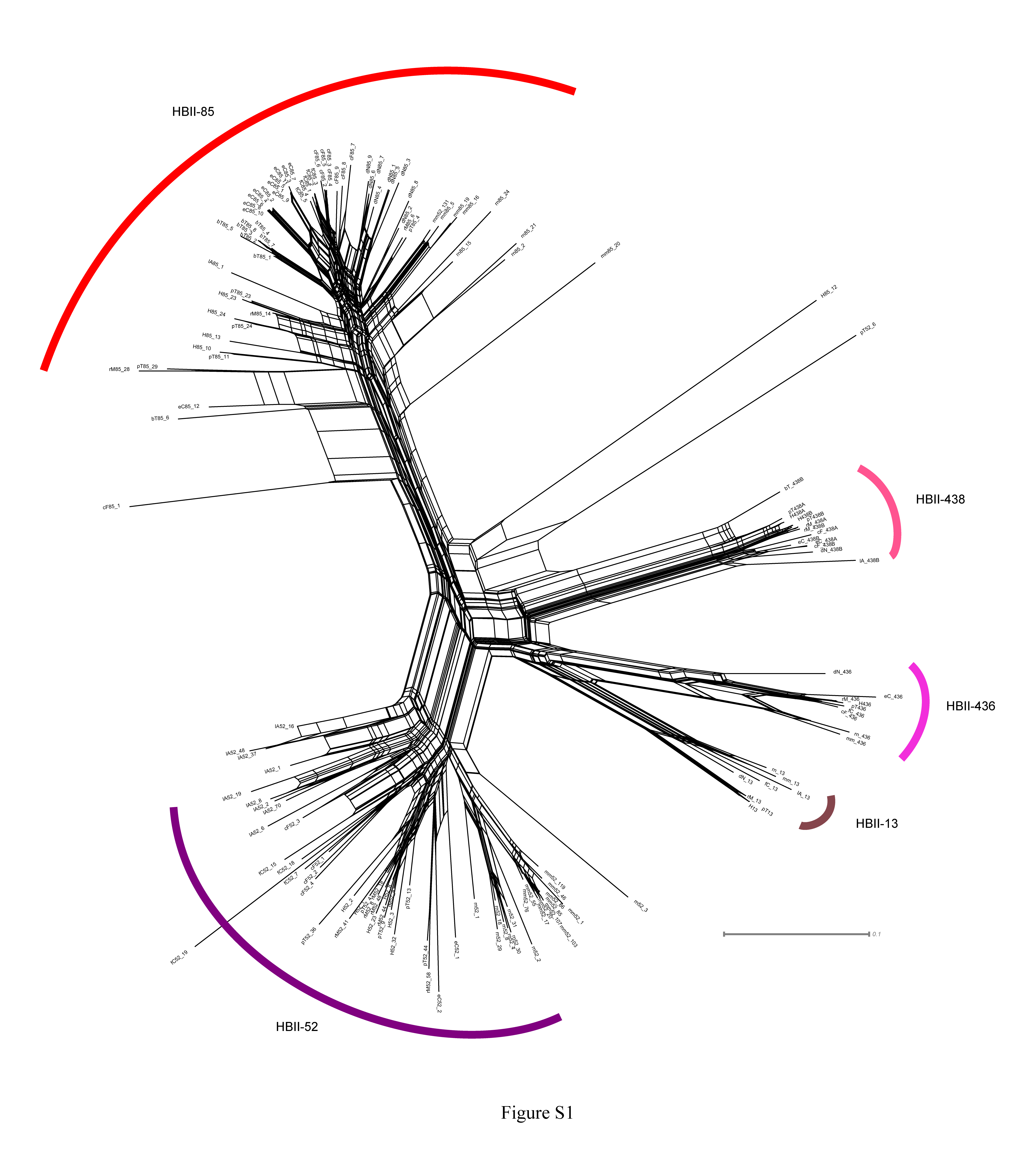

Supplement: Figure S1 — Phylogenetic network of imprinted snoRNAs in the PWS locus. Gene families were indicted with different colors. Species abbreviations: H, human; MM, mouse; RN, rat; CF, dog; BT, cow; DN, armadillo; LA, elephant. (TIF) [file pone.0100329.s001.tif]

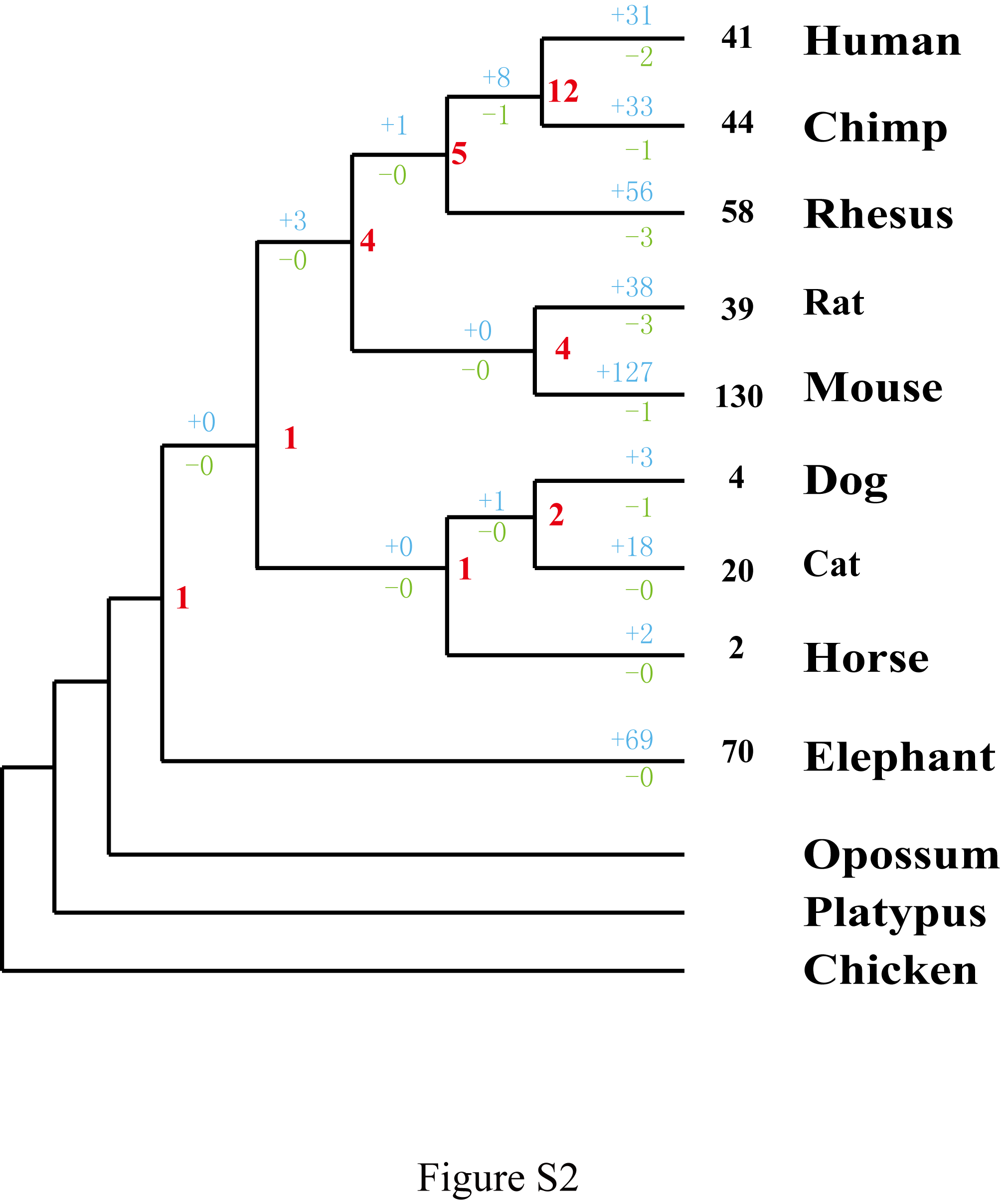

Supplement: Figure S2 — Gene numbers and changes in in the HBII-52 gene family. Numbers in black indicate the current gene number in each species, whereas red numbers indicate the ancestral gene number, blue numbers denote gained genes, and green numbers indicate lost genes. (TIF) [file pone.0100329.s002.tif]

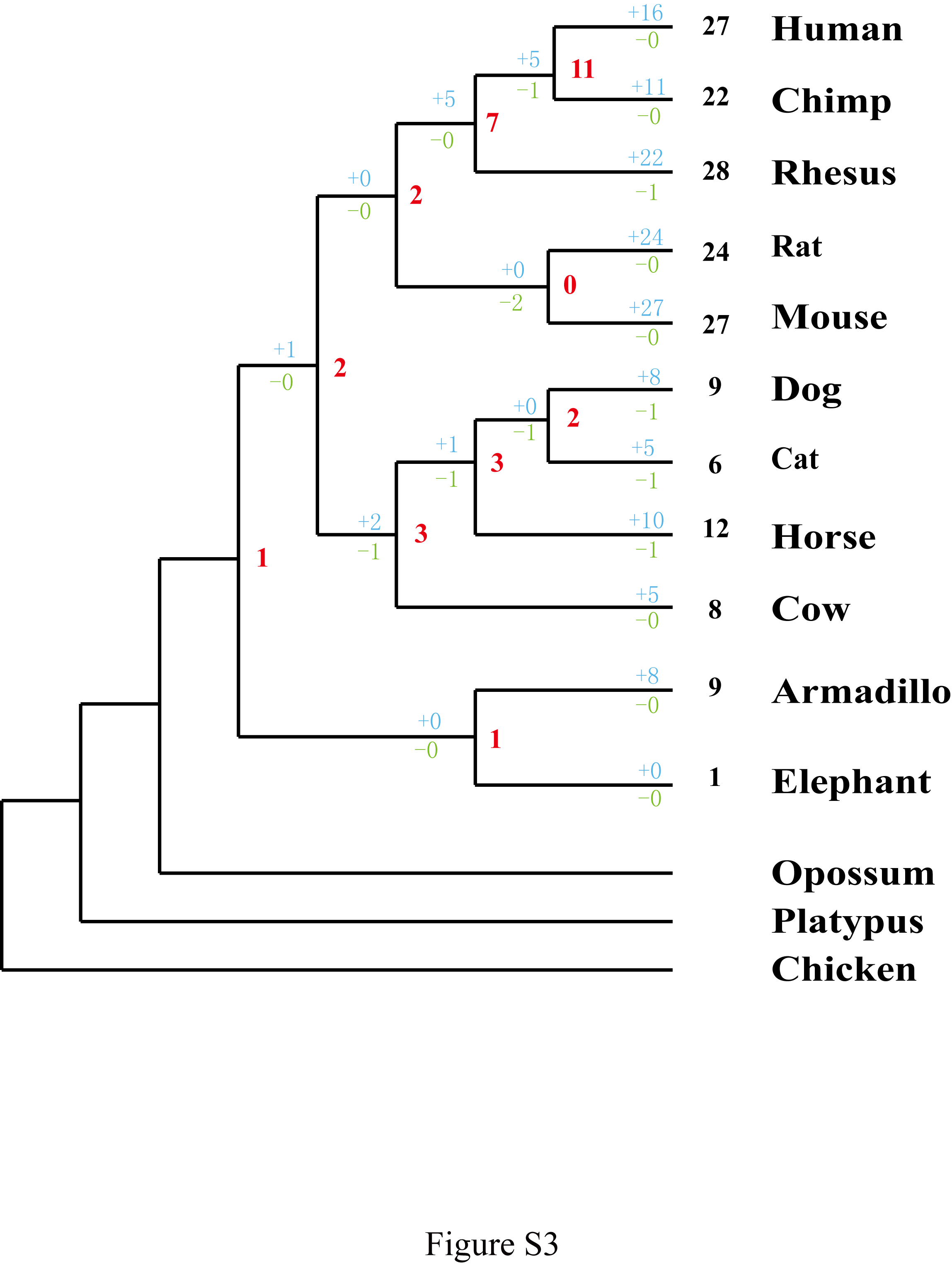

Supplement: Figure S3 — Gene numbers and changes in in the HBII-85 gene family. (TIF) [file pone.0100329.s003.tif]

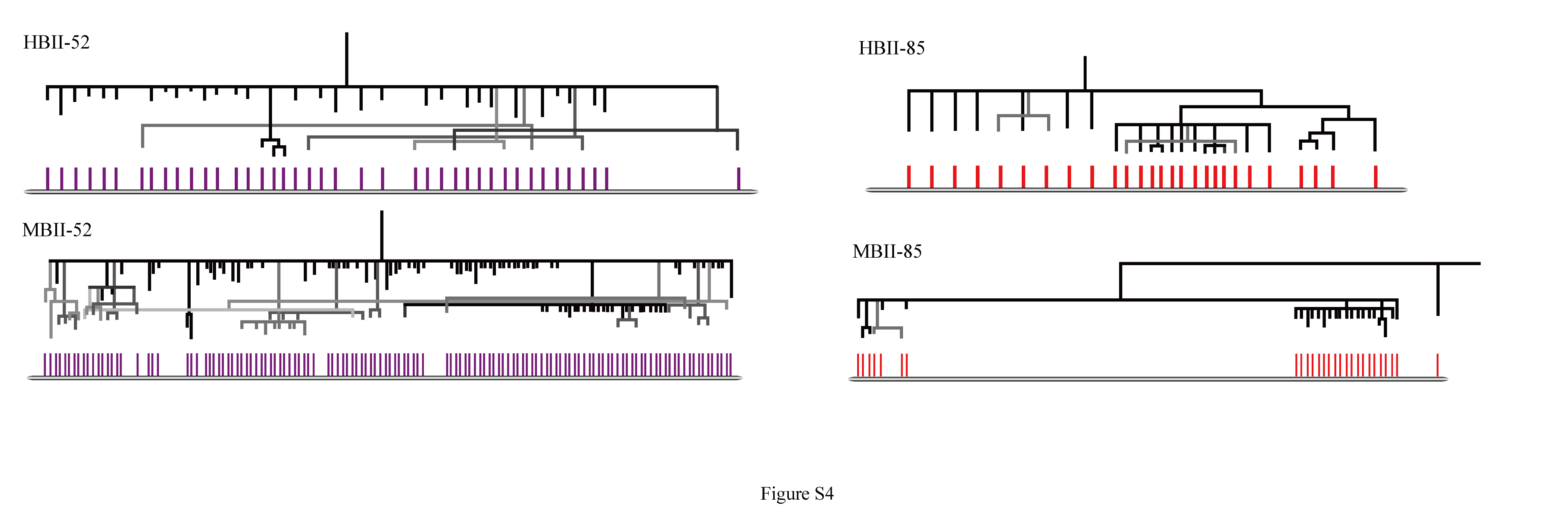

Supplement: Figure S4 — Combined physical-phylogenetic map. Imprinted snoRNA genes are depicted as vertical bar as in figure 1. The consistency between phylogenetic relationship and physical distribution suggest the genes are created by tandem duplication. (TIF) [file pone.0100329.s004.tif]

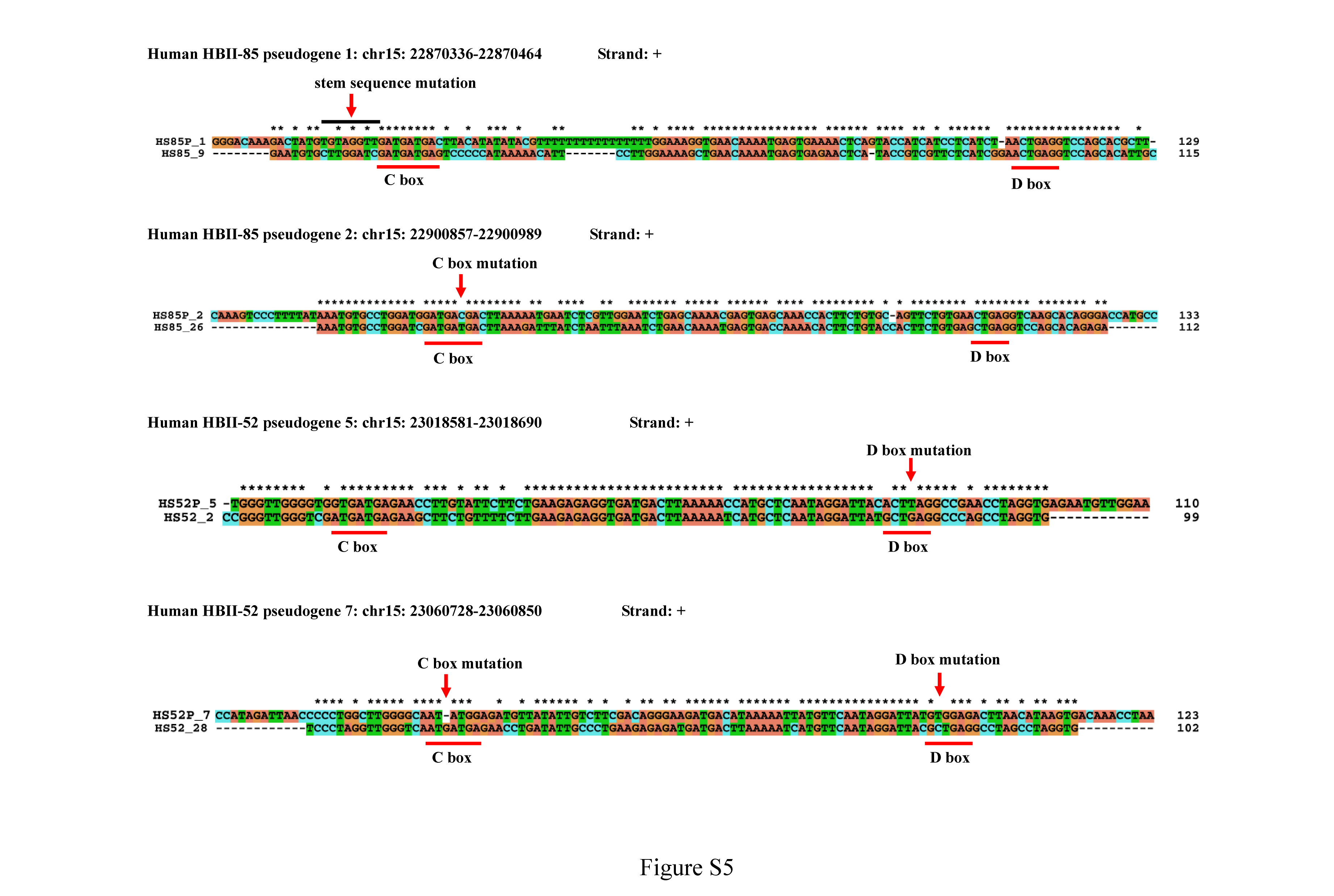

Supplement: Figure S5 — Human HBII-85 and HBII-52 pseudogenes. Selected pseudogenes were aligned with corresponding normal genes. Red arrow indicate loss-of-function mutations in these pseudo genes. (TIF) [file pone.0100329.s005.tif]

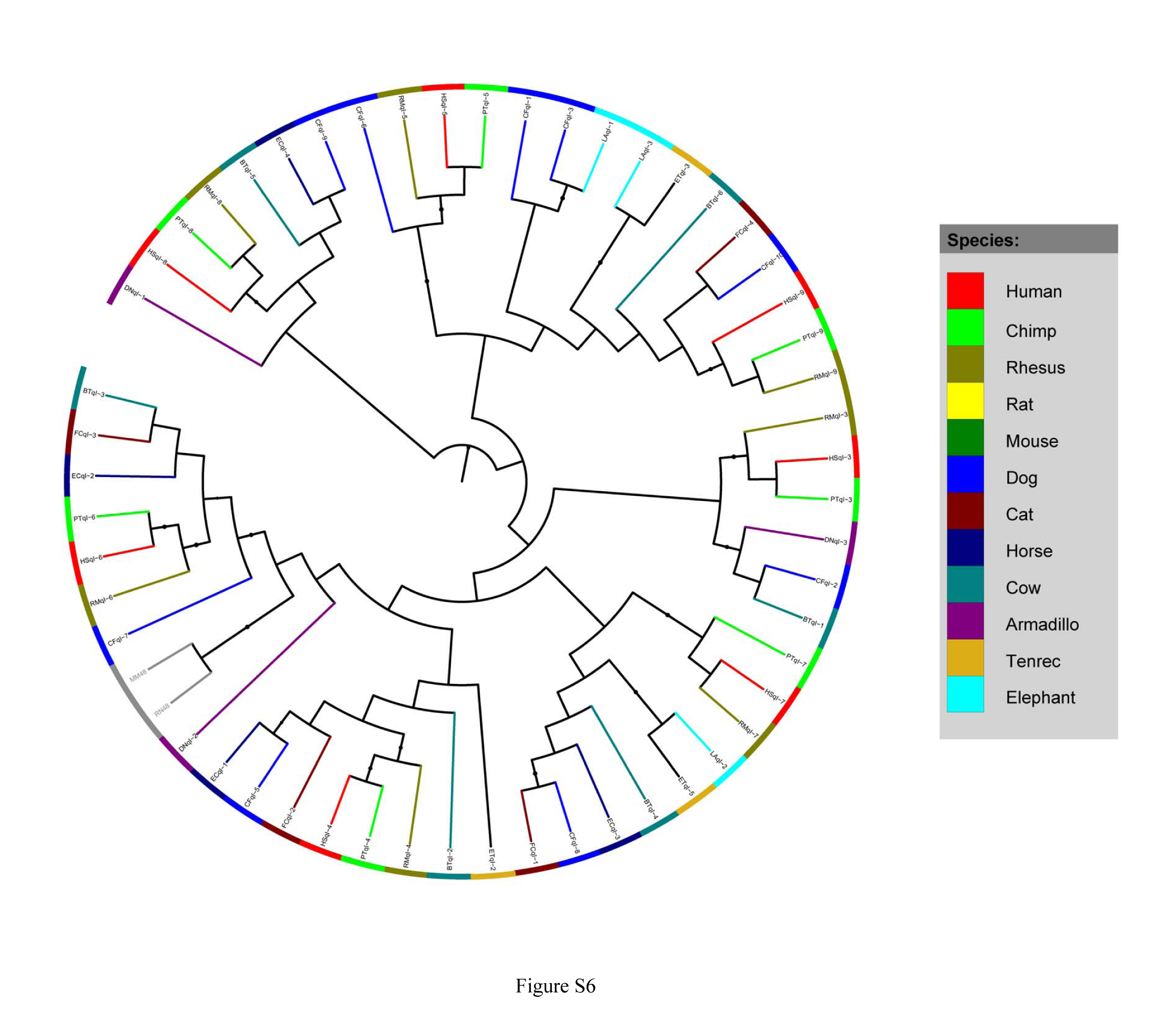

Supplement: Figure S6 — Phylogenetic analysis of 14q(I) gene family. Abbreviations: HS, human; PT, chimpanzee; RM, rhesus; MM, mouse; RN, rat; CF, dog; FC, cat; EC, horse; BT, cow; DN, armadillo; LA, elephant. (TIF) [file pone.0100329.s006.tif]
