# Supplementary material for: Rapid Birth-and-Death Evolution of Imprinted snoRNAs in the Prader-Willi Syndrome Locus: Implications for Neural Development in Euarchontoglires
Source: PLoS One. 2014 Jun 19;9(6):e100329. doi: 10.1371/journal.pone.0100329 (PMC4063771; doi:10.1371/journal.pone.0100329)
Supplement: Table S1 — Chromosome loci, size, and the genomic position of PWS imprinted regions in 12 eutherians. (PDF) [file pone.0100329.s007.pdf]

Table S1 Chromosome loci, size, and the genomic position of *PWS* imprinted regions in 12 eutherians.

Species	Genome size (Gb)	*PWS* imprinted region		
		Chromosome loci, scaffolds	Size (kb)	Sequence range
**Human** (*Homo sapiens*) [hg18]	2.8	15q11q13	2297.5	chr15:21351547-25461729
**Chimp** (*Pan troglodytes*) [panTro2]	2.8	Chr15	2238.9	chr15:21048659-25178235
**Rhesus** (*Macaca mulatta*) [rheMac2]		Chr7	1973.3	chr7:2748701-6486499
**Rat** (*Rattus norvegicus*) [rn4]	2.7	Chr1	6499.8	chr1:108139311-116725368
**Mouse** (*Mus musculus*) [mm8]	2.5	Chr7	3772.5	chr7:57140856-62349089
**Dog** (*Canis lupus familiaris*) [canFam2]	2.4	Chr3	1586.5	chr3:35800947-39289249
**Cat** (*Felis catus*) [felCat3]		Scaffolds	ND	scaffold_213295:22600-218526<br>scaffold_213296:30-146699<br>scaffold_147394:36378-275129 …
**Horse** (*Equus caballus*) [equCab1]		Chr1	1645.4	chr1:106821552-110271319
**Cow** (*Bos taurus*) [bosTau3]	2.4	Chr21	ND	chr21:2492391-3783054<br>chr21:6110944-7260509<br>chrUn.003.36:119-311795 …
**Armadillo** (*Dasypus novemcinctus*) [dasNov1]	~3.0	Scaffolds	ND	scaffold_24054:3-24086<br>scaffold_4945:40116-129444<br>scaffold_32582:3-13801…
**Tenrec** (*Echinops telfairi*) [echTel1]	~3.0	Scaffolds	ND	scaffold_252512:2435-10387<br>scaffold_280835:6667-53483<br>scaffold_302458:63-10234...
**Elephant** (*Loxodonta africana*) [loxAfr1]	~3.0	Scaffolds	ND	scaffold_21049:5-28748<br>scaffold_4282:1638-79325<br>scaffold_11475:2007-30261 …

[ ], the UCSC genome version used in this study.
