# Supplementary material for: Rapid Birth-and-Death Evolution of Imprinted snoRNAs in the Prader-Willi Syndrome Locus: Implications for Neural Development in Euarchontoglires
Source: PLoS One. 2014 Jun 19;9(6):e100329. doi: 10.1371/journal.pone.0100329 (PMC4063771; doi:10.1371/journal.pone.0100329)
Supplement: Table S2 — Pseudogenes identified in the PWS imprinted region. (PDF) [file pone.0100329.s008.pdf]

Table S2. Conserved non-imprinted box C/D snoRNA gene families between human and other species.

human-chimp (105)	human-rhesus (101)	human-mouse (103)	human-rat (103)	human-dog (105)	human-cat (93)	human-cow (101)	human-horse (105)	human-armadillo (78)	human-tenrec (87)	human-elephant (72)	human-opposum (97)	human-platypus (91)	human-chicken (77)
SNORD1	SNORD1	SNORD1	SNORD1	SNORD1	SNORD1	SNORD1	SNORD1	SNORD1	SNORD1	SNORD4	SNORD1	SNORD1	SNORD1
SNORD2	SNORD2	SNORD2	SNORD2	SNORD2	SNORD5	SNORD2	SNORD2	SNORD2	SNORD2	SNORD7	SNORD2	SNORD2	SNORD2
SNORD4	SNORD4	SNORD4	SNORD4	SNORD4	SNORD6	SNORD5	SNORD4	SNORD4	SNORD4	SNORD8	SNORD4	SNORD4	SNORD4
SNORD5	SNORD5	SNORD5	SNORD5	SNORD5	SNORD7	SNORD6	SNORD5	SNORD5	SNORD5	SNORD10	SNORD5	SNORD5	SNORD5
SNORD6	SNORD6	SNORD6	SNORD6	SNORD6	SNORD8	SNORD7	SNORD6	SNORD6	SNORD6	SNORD11	SNORD6	SNORD6	SNORD6
SNORD7	SNORD7	SNORD7	SNORD7	SNORD7	SNORD10	SNORD8	SNORD7	SNORD8	SNORD7	SNORD12	SNORD7	SNORD7	SNORD7
SNORD8	SNORD8	SNORD8	SNORD8	SNORD8	SNORD11	SNORD10	SNORD8	SNORD11	SNORD8	SNORD15	SNORD8	SNORD11	SNORD11
SNORD10	SNORD10	SNORD10	SNORD10	SNORD10	SNORD12	SNORD11	SNORD10	SNORD12	SNORD10	SNORD16	SNORD10	SNORD12	SNORD12
SNORD11	SNORD11	SNORD11	SNORD11	SNORD11	SNORD14	SNORD12	SNORD11	SNORD14	SNORD11	SNORD18	SNORD11	SNORD14	SNORD14
SNORD12	SNORD12	SNORD12	SNORD12	SNORD12	SNORD15	SNORD14	SNORD12	SNORD15	SNORD12	SNORD19	SNORD12	SNORD16	SNORD15
SNORD14	SNORD14	SNORD14	SNORD14	SNORD14	SNORD16	SNORD15	SNORD14	SNORD16	SNORD14	SNORD20	SNORD14	SNORD17	SNORD16
SNORD15	SNORD15	SNORD15	SNORD15	SNORD15	SNORD17	SNORD16	SNORD15	SNORD18	SNORD15	SNORD21	SNORD15	SNORD18	SNORD17
SNORD16	SNORD16	SNORD16	SNORD16	SNORD16	SNORD18	SNORD17	SNORD16	SNORD19	SNORD16	SNORD24	SNORD16	SNORD19	SNORD18
SNORD17	SNORD17	SNORD17	SNORD17	SNORD17	SNORD19	SNORD18	SNORD17	SNORD20	SNORD18	SNORD30	SNORD17	SNORD20	SNORD19
SNORD18	SNORD18	SNORD18	SNORD18	SNORD18	SNORD24	SNORD19	SNORD18	SNORD23	SNORD19	SNORD31	SNORD18	SNORD21	SNORD20
SNORD19	SNORD19	SNORD19	SNORD19	SNORD19	SNORD25	SNORD20	SNORD19	SNORD25	SNORD20	SNORD32	SNORD19	SNORD24	SNORD21
SNORD20	SNORD21	SNORD20	SNORD20	SNORD20	SNORD26	SNORD21	SNORD20	SNORD26	SNORD21	SNORD33	SNORD20	SNORD25	SNORD24
SNORD21	SNORD23	SNORD21	SNORD21	SNORD21	SNORD28	SNORD23	SNORD21	SNORD27	SNORD23	SNORD34	SNORD21	SNORD27	SNORD32
SNORD23	SNORD24	SNORD23	SNORD23	SNORD23	SNORD31	SNORD24	SNORD23	SNORD28	SNORD25	SNORD36	SNORD24	SNORD28	SNORD35
SNORD24	SNORD25	SNORD24	SNORD24	SNORD24	SNORD33	SNORD25	SNORD24	SNORD29	SNORD26	SNORD38	SNORD26	SNORD29	SNORD36
SNORD25	SNORD26	SNORD25	SNORD25	SNORD25	SNORD34	SNORD26	SNORD25	SNORD30	SNORD32	SNORD42	SNORD27	SNORD30	SNORD37
SNORD26	SNORD27	SNORD26	SNORD26	SNORD26	SNORD35	SNORD27	SNORD26	SNORD31	SNORD33	SNORD43	SNORD28	SNORD31	SNORD38
SNORD27	SNORD28	SNORD27	SNORD27	SNORD27	SNORD36	SNORD28	SNORD27	SNORD35	SNORD34	SNORD45	SNORD29	SNORD32	SNORD42
SNORD28	SNORD29	SNORD28	SNORD28	SNORD28	SNORD37	SNORD29	SNORD28	SNORD36	SNORD35	SNORD47	SNORD30	SNORD33	SNORD43
SNORD29	SNORD30	SNORD29	SNORD29	SNORD29	SNORD38	SNORD30	SNORD29	SNORD37	SNORD36	SNORD48	SNORD31	SNORD34	SNORD44
SNORD30	SNORD31	SNORD30	SNORD30	SNORD30	SNORD41	SNORD31	SNORD30	SNORD38	SNORD37	SNORD50	SNORD32	SNORD35	SNORD45
SNORD31	SNORD32	SNORD31	SNORD31	SNORD31	SNORD42	SNORD32	SNORD31	SNORD41	SNORD38	SNORD51	SNORD33	SNORD36	SNORD46
SNORD32	SNORD33	SNORD32	SNORD32	SNORD32	SNORD43	SNORD33	SNORD32	SNORD42	SNORD41	SNORD52	SNORD34	SNORD37	SNORD47

SNORD33	SNORD34	SNORD33	SNORD33	SNORD33	SNORD44	SNORD34	SNORD33	SNORD44	SNORD42	SNORD54	SNORD36	SNORD42	SNORD51
SNORD34	SNORD35	SNORD34	SNORD34	SNORD34	SNORD45	SNORD35	SNORD34	SNORD46	SNORD43	SNORD55	SNORD37	SNORD43	SNORD53
SNORD35	SNORD36	SNORD35	SNORD35	SNORD35	SNORD48	SNORD36	SNORD35	SNORD47	SNORD44	SNORD57	SNORD38	SNORD44	SNORD54
SNORD36	SNORD37	SNORD36	SNORD36	SNORD36	SNORD49	SNORD37	SNORD36	SNORD49	SNORD45	SNORD58	SNORD41	SNORD45	SNORD55
SNORD37	SNORD38	SNORD37	SNORD37	SNORD37	SNORD50	SNORD38	SNORD37	SNORD50	SNORD47	SNORD60	SNORD42	SNORD47	SNORD57
SNORD38	SNORD41	SNORD38	SNORD38	SNORD38	SNORD52	SNORD41	SNORD38	SNORD52	SNORD48	SNORD61	SNORD44	SNORD49	SNORD58
SNORD41	SNORD42	SNORD41	SNORD41	SNORD41	SNORD53	SNORD43	SNORD41	SNORD55	SNORD49	SNORD62	SNORD45	SNORD50	SNORD59
SNORD42	SNORD43	SNORD42	SNORD42	SNORD42	SNORD54	SNORD44	SNORD42	SNORD56	SNORD52	SNORD65	SNORD46	SNORD51	SNORD60
SNORD43	SNORD44	SNORD43	SNORD43	SNORD43	SNORD55	SNORD45	SNORD43	SNORD58	SNORD53	SNORD68	SNORD47	SNORD53	SNORD61
SNORD44	SNORD45	SNORD44	SNORD44	SNORD44	SNORD56	SNORD46	SNORD44	SNORD59	SNORD54	SNORD70	SNORD48	SNORD54	SNORD65
SNORD45	SNORD46	SNORD45	SNORD45	SNORD45	SNORD57	SNORD47	SNORD45	SNORD60	SNORD55	SNORD72	SNORD49	SNORD55	SNORD66
SNORD46	SNORD47	SNORD46	SNORD46	SNORD46	SNORD58	SNORD48	SNORD46	SNORD61	SNORD58	SNORD73	SNORD50	SNORD56	SNORD67
SNORD47	SNORD48	SNORD47	SNORD47	SNORD47	SNORD59	SNORD49	SNORD47	SNORD62	SNORD59	SNORD74	SNORD51	SNORD57	SNORD68
SNORD48	SNORD49	SNORD48	SNORD48	SNORD48	SNORD60	SNORD50	SNORD48	SNORD63	SNORD62	SNORD75	SNORD52	SNORD58	SNORD69
SNORD49	SNORD50	SNORD49	SNORD49	SNORD49	SNORD61	SNORD51	SNORD49	SNORD65	SNORD63	SNORD76	SNORD53	SNORD59	SNORD70
SNORD50	SNORD51	SNORD50	SNORD50	SNORD50	SNORD62	SNORD52	SNORD50	SNORD67	SNORD65	SNORD77	SNORD54	SNORD60	SNORD71
SNORD51	SNORD52	SNORD51	SNORD51	SNORD51	SNORD63	SNORD53	SNORD51	SNORD68	SNORD67	SNORD80	SNORD56	SNORD61	SNORD72
SNORD52	SNORD53	SNORD52	SNORD52	SNORD52	SNORD65	SNORD54	SNORD52	SNORD69	SNORD68	SNORD81	SNORD57	SNORD62	SNORD73
SNORD53	SNORD54	SNORD53	SNORD53	SNORD53	SNORD66	SNORD55	SNORD53	SNORD70	SNORD69	SNORD82	SNORD58	SNORD63	SNORD74
SNORD54	SNORD55	SNORD54	SNORD54	SNORD54	SNORD67	SNORD56	SNORD54	SNORD73	SNORD70	SNORD84	SNORD59	SNORD65	SNORD75
SNORD55	SNORD56	SNORD55	SNORD55	SNORD55	SNORD68	SNORD57	SNORD56	SNORD74	SNORD71	SNORD86	SNORD60	SNORD66	SNORD76
SNORD56	SNORD57	SNORD56	SNORD56	SNORD56	SNORD70	SNORD58	SNORD57	SNORD75	SNORD72	SNORD87	SNORD61	SNORD67	SNORD77
SNORD57	SNORD58	SNORD57	SNORD57	SNORD57	SNORD71	SNORD59	SNORD58	SNORD76	SNORD73	SNORD88	SNORD62	SNORD68	SNORD79
SNORD58	SNORD59	SNORD58	SNORD58	SNORD58	SNORD72	SNORD60	SNORD59	SNORD77	SNORD74	SNORD90	SNORD65	SNORD69	SNORD82
SNORD59	SNORD60	SNORD59	SNORD59	SNORD59	SNORD73	SNORD62	SNORD60	SNORD78	SNORD75	SNORD92	SNORD66	SNORD70	SNORD83
SNORD60	SNORD62	SNORD60	SNORD60	SNORD60	SNORD75	SNORD63	SNORD61	SNORD79	SNORD76	SNORD93	SNORD67	SNORD71	SNORD84
SNORD61	SNORD63	SNORD61	SNORD61	SNORD61	SNORD76	SNORD65	SNORD62	SNORD80	SNORD78	SNORD95	SNORD68	SNORD72	SNORD86
SNORD62	SNORD65	SNORD62	SNORD62	SNORD62	SNORD77	SNORD66	SNORD63	SNORD81	SNORD79	SNORD96	SNORD69	SNORD73	SNORD87
SNORD63	SNORD66	SNORD63	SNORD63	SNORD63	SNORD78	SNORD67	SNORD65	SNORD82	SNORD80	SNORD98	SNORD70	SNORD74	SNORD88
SNORD65	SNORD67	SNORD65	SNORD65	SNORD65	SNORD79	SNORD68	SNORD66	SNORD84	SNORD81	SNORD99	SNORD71	SNORD75	SNORD89
SNORD66	SNORD68	SNORD66	SNORD66	SNORD66	SNORD80	SNORD69	SNORD67	SNORD86	SNORD82	SNORD102	SNORD72	SNORD76	SNORD90
SNORD67	SNORD69	SNORD67	SNORD67	SNORD67	SNORD83	SNORD70	SNORD68	SNORD87	SNORD83	SNORD103	SNORD73	SNORD77	SNORD91

SNORD68	SNORD70	SNORD68	SNORD68	SNORD68	SNORD84	SNORD71	SNORD69	SNORD88	SNORD84	SNORD110	SNORD74	SNORD78	SNORD92
SNORD69	SNORD71	SNORD69	SNORD69	SNORD69	SNORD86	SNORD72	SNORD70	SNORD89	SNORD86	SNORD111	SNORD75	SNORD79	SNORD93
SNORD70	SNORD72	SNORD70	SNORD70	SNORD70	SNORD87	SNORD73	SNORD71	SNORD90	SNORD87	SNORD64	SNORD76	SNORD80	SNORD94
SNORD71	SNORD73	SNORD71	SNORD71	SNORD71	SNORD89	SNORD74	SNORD72	SNORD91	SNORD88	SNORD109	SNORD77	SNORD81	SNORD95
SNORD72	SNORD74	SNORD72	SNORD72	SNORD72	SNORD90	SNORD75	SNORD73	SNORD92	SNORD91	SNORD113	SNORD78	SNORD82	SNORD98
SNORD73	SNORD76	SNORD73	SNORD73	SNORD73	SNORD91	SNORD76	SNORD74	SNORD94	SNORD92	SNORD114	SNORD79	SNORD83	SNORD99
SNORD74	SNORD77	SNORD74	SNORD74	SNORD74	SNORD92	SNORD78	SNORD75	SNORD95	SNORD94	SNORD115	SNORD80	SNORD84	SNORD100
SNORD75	SNORD78	SNORD75	SNORD75	SNORD75	SNORD93	SNORD79	SNORD76	SNORD100	SNORD95	SNORD116	SNORD81	SNORD87	SNORD102
SNORD76	SNORD79	SNORD76	SNORD76	SNORD76	SNORD94	SNORD80	SNORD77	SNORD102	SNORD96	SNORD119	SNORD82	SNORD88	SNORD103
SNORD77	SNORD80	SNORD78	SNORD78	SNORD77	SNORD95	SNORD81	SNORD78	SNORD103	SNORD98	SNORD121	SNORD83	SNORD89	SNORD110
SNORD78	SNORD81	SNORD79	SNORD79	SNORD78	SNORD96	SNORD82	SNORD79	SNORD104	SNORD99	SNORD124	SNORD84	SNORD90	SNORD111
SNORD79	SNORD83	SNORD80	SNORD80	SNORD79	SNORD98	SNORD83	SNORD80	SNORD105	SNORD100	SNORD127	SNORD86	SNORD91	SNORD121
SNORD80	SNORD84	SNORD81	SNORD81	SNORD80	SNORD99	SNORD84	SNORD81	SNORD110	SNORD102		SNORD87	SNORD92	SNORD123
SNORD81	SNORD86	SNORD82	SNORD82	SNORD81	SNORD100	SNORD86	SNORD82	SNORD111	SNORD103		SNORD88	SNORD93	SNORD124
SNORD82	SNORD87	SNORD83	SNORD83	SNORD82	SNORD103	SNORD87	SNORD83	SNORD117	SNORD104		SNORD89	SNORD94	SNORD125
SNORD83	SNORD88	SNORD84	SNORD84	SNORD83	SNORD104	SNORD88	SNORD84	SNORD121	SNORD105		SNORD90	SNORD95	SNORD127
SNORD84	SNORD89	SNORD86	SNORD86	SNORD86	SNORD105	SNORD89	SNORD86	SNORD126	SNORD110		SNORD91	SNORD96	SNORD130
SNORD86	SNORD90	SNORD87	SNORD87	SNORD87	SNORD110	SNORD90	SNORD87	SNORD130	SNORD111		SNORD92	SNORD98	
SNORD87	SNORD91	SNORD88	SNORD88	SNORD88	SNORD111	SNORD91	SNORD88		SNORD117		SNORD93	SNORD99	
SNORD88	SNORD92	SNORD89	SNORD89	SNORD89	SNORD64	SNORD92	SNORD89		SNORD119		SNORD94	SNORD100	
SNORD89	SNORD93	SNORD90	SNORD90	SNORD90	SNORD107	SNORD93	SNORD90		SNORD121		SNORD95	SNORD102	
SNORD90	SNORD94	SNORD91	SNORD91	SNORD91	SNORD113	SNORD94	SNORD91		SNORD123		SNORD96	SNORD103	
SNORD92	SNORD95	SNORD92	SNORD92	SNORD92	SNORD114	SNORD95	SNORD92		SNORD124		SNORD98	SNORD104	
SNORD93	SNORD96	SNORD93	SNORD93	SNORD93	SNORD115	SNORD96	SNORD93		SNORD125		SNORD99	SNORD105	
SNORD94	SNORD98	SNORD94	SNORD94	SNORD94	SNORD116	SNORD98	SNORD94		SNORD126		SNORD100	SNORD111	
SNORD95	SNORD99	SNORD95	SNORD95	SNORD95	SNORD117	SNORD99	SNORD95		SNORD127		SNORD102	SNORD121	
SNORD96	SNORD100	SNORD96	SNORD96	SNORD96	SNORD119	SNORD100	SNORD96		SNORD130		SNORD103	SNORD123	
SNORD98	SNORD102	SNORD98	SNORD98	SNORD98	SNORD121	SNORD102	SNORD98				SNORD104	SNORD125	
SNORD99	SNORD103	SNORD99	SNORD100	SNORD99	SNORD123	SNORD103	SNORD99				SNORD105	SNORD126	
SNORD100	SNORD104	SNORD100	SNORD102	SNORD100	SNORD124	SNORD104	SNORD100				SNORD111	SNORD127	
SNORD102	SNORD105	SNORD102	SNORD103	SNORD102	SNORD125	SNORD105	SNORD102				SNORD117	SNORD130	
SNORD103	SNORD111	SNORD103	SNORD104	SNORD103	SNORD126	SNORD110	SNORD103				SNORD119		

SNORD104	SNORD117	SNORD104	SNORD105	SNORD104	SNORD130	SNORD111	SNORD104				SNORD121
SNORD105	SNORD119	SNORD105	SNORD110	SNORD105		SNORD117	SNORD105				SNORD123
SNORD110	SNORD121	SNORD110	SNORD111	SNORD110		SNORD119	SNORD110				SNORD126
SNORD111	SNORD123	SNORD111	SNORD117	SNORD111		SNORD121	SNORD111				SNORD127
SNORD117	SNORD124	SNORD117	SNORD119	SNORD117		SNORD123	SNORD117				SNORD130
SNORD119	SNORD125	SNORD119	SNORD121	SNORD119		SNORD124	SNORD119				
SNORD121	SNORD126	SNORD121	SNORD123	SNORD121		SNORD125	SNORD121				
SNORD123	SNORD127	SNORD123	SNORD124	SNORD123		SNORD127	SNORD123				
SNORD124	SNORD130	SNORD124	SNORD126	SNORD124		SNORD130	SNORD124				
SNORD125		SNORD127	SNORD127	SNORD125			SNORD125				
SNORD126		SNORD130	SNORD130	SNORD126			SNORD126				
SNORD127				SNORD127			SNORD127				
SNORD130				SNORD130			SNORD130				

(), number of gene families conserved between human and other species; □, orphan snoRNA gene.
