# Supplementary material for: Rapid Birth-and-Death Evolution of Imprinted snoRNAs in the Prader-Willi Syndrome Locus: Implications for Neural Development in Euarchontoglires
Source: PLoS One. 2014 Jun 19;9(6):e100329. doi: 10.1371/journal.pone.0100329 (PMC4063771; doi:10.1371/journal.pone.0100329)
Supplement: Table S3 — Conserved non-imprinted box C/D snoRNA gene families between human and other species. (PDF) [file pone.0100329.s009.pdf]

Table S3 Pseudogenes identified in the PWS imprinted region.

**Lineages**	Species	HBII-85 gene family				HBII-52 gene family			
		Genes	Pseudogenes	Ratio of pseudogenes/ total genes	Mean ratio of lineage	Genes	Pseudogenes	Ratio of pseudogenes/ total genes	Mean ratio of lineage
**Primates**	Human	27	2	0.069	0.136	41	9	0.180	0.138
	Chimp	22	6	0.214		44	8	0.154	
	Rhesus	28	4	0.125		58	5	0.079	
**Rodentia**	Mouse	27	0	0.0	0.147	130	9	0.065	0.158
	Rat	24	10	0.294		39	13	0.250	
**Laurasiatheri**	Dog	9	6	0.400	0.256	4	2	0.333	0.667
	Cow	8	1	0.111		0	5	1.000	

Note：Ratio of pseudogenes is caculated by pseudogenes/(genes+ pseudogenes).
