# Supplementary material for: Rapid Birth-and-Death Evolution of Imprinted snoRNAs in the Prader-Willi Syndrome Locus: Implications for Neural Development in Euarchontoglires
Source: PLoS One. 2014 Jun 19;9(6):e100329. doi: 10.1371/journal.pone.0100329 (PMC4063771; doi:10.1371/journal.pone.0100329)
Supplement: Table S4 — Nucleotide divergence between human and other species for non-imprinted and imprinted snoRNA genes. (PDF) [file pone.0100329.s010.pdf]

Table S4. Nucleotide divergence between human and other species for non-imprinted and imprinted snoRNA genes.

		Human vs.													
	Species	chimp	rhesus	mouse	rat	dog	cat	horse	cow	armadillo	elephant	tenrec	opossum	platypus	chicken
Non-imprinted snoRNAs	Divergence	0.043	0.046	0.188	0.18	0.153	0.174	0.142	0.148	0.174	0.151	0.195	0.295	0.309	0.381
	S.E	0.002	0.002	0.004	0.004	0.005	0.005	0.005	0.005	0.006	0.005	0.004	0.007	0.006	0.009
Imprinted HBII-52 snoRNAs	Divergence	0.085	0.073	0.222	0.265	0.248	0.268	0.249	-	-	0.331	-	-	-	-
	S.E	-0.001	0	0.164	0.193	0.132	0.155	0.071			0.197				
	Relative divergence (imprinted/non-imprinted)	*1.98*	*1.59*	*1.18*	*1.47*	*1.62*	*1.54*	*1.75*			*2.19*				
Imprinted HBII-85 snoRNAs	Divergence	0.2	0.185	0.251	0.242	0.277	0.358	0.254	0.316	0.258	0.234	–	–	–	–
	S.E	0.029	0.026	0.041	0.038	0.037	0.042	0.038	0.039	0.037	0.041				
	Relative divergence (imprinted/non-imprinted)	*4.65*	*4.02*	*1.34*	*1.34*	*1.81*	*2.06*	*1.79*	*2.14*	*1.48*	*1.55*				

S.E., standard error; -, the gene family was not detected.
