# Supplementary material for: Rapid Birth-and-Death Evolution of Imprinted snoRNAs in the Prader-Willi Syndrome Locus: Implications for Neural Development in Euarchontoglires
Source: PLoS One. 2014 Jun 19;9(6):e100329. doi: 10.1371/journal.pone.0100329 (PMC4063771; doi:10.1371/journal.pone.0100329)
Supplement: Table S5 — Intra- and inter- genomic divergence for PWS-related imprinted snoRNA genes. (PDF) [file pone.0100329.s011.pdf]

Table S5. Intra- and inter- genomic divergence for PWS-related imprinted snoRNA genes.

	HBII-85	HBII-52
Intra-genomic divergenece	0.132 (0.015)	0.133 (0.012)
Inter-genomic divergenece	0.100 (0.016)	0.096 (0.026)

(), standard deviation.
